# Myc-driven chromatin accessibility regulates Cdc45 assembly into CMG helicases

**DOI:** 10.1038/s42003-019-0353-2

**Published:** 2019-03-22

**Authors:** Brook S. Nepon-Sixt, Victoria L. Bryant, Mark G. Alexandrow

**Affiliations:** 1 Department of Molecular Oncology, Moffitt Cancer Center and Research Institute, Tampa, FL 33612 USA; 2 University of South Florida Cancer Biology PhD Program, Tampa, FL 33612 USA; 3 Present Address: AT Still University School of Osteopathic Medicine 27 5850 E Still Circle, Mesa, AZ 85206 USA

## Abstract

Myc-driven tumorigenesis involves a non-transcriptional role for Myc in over-activating replication origins. We show here that the mechanism underlying this process involves a direct role for Myc in activation of Cdc45-MCM-GINS (CMG) helicases at Myc-targeted sites. Myc induces decondensation of higher-order chromatin at targeted sites and is required for chromatin access at a chromosomal origin. Myc-driven chromatin accessibility promotes Cdc45/GINS recruitment to resident MCMs, and activation of CMGs. Myc-Box II, which is necessary for Myc-driven transformation, is required for Myc-induced chromatin accessibility, Cdc45/GINS recruitment, and replication stimulation. Myc interactors GCN5, Tip60, and TRRAP are essential for chromatin unfolding and recruitment of Cdc45, and co-expression of GCN5 or Tip60 with MBII-deficient Myc rescues these events and promotes CMG activation. Finally, Myc and Cdc45 interact and physiologic conditions for CMG assembly require the functions of Myc, MBII, and GCN5 for Cdc45 recruitment and initiation of DNA replication.

## Abstract (web summary)

Nepon-Sixt, Bryant, and Alexandrow show that Myc increases the chromatin accessibility through Myc-Box II, activating Cdc45-MCM-GINS helicases at Myc-targeted sites. This study highlights a non-transcriptional role for Myc in over-activating DNA replication, providing insight into Myc-driven tumorigenesis.

## Introduction

The family of *myc* proto-oncogenes (c-, N-, and L-*myc*) encode essential proteins (called Myc) that regulate expression of genes controlling cellular growth, differentiation, and metabolism^1^. Myc is often overexpressed in human cancers, and promotes tumorigenesis in part by altering the transcriptome^2–4^. However, Myc is atypical relative to canonical transcriptional regulators. Myc is only modest in magnitude at altering transcription of target genes, and Myc influences thousands of targets in addition to specific gene regulation^5,6^. Myc interacts with vast regions of chromosomes, often at great distances from promoters and throughout intergenic regions. Myc appears to interact with more than 25,000 binding sites in the genome, almost half being >10 kb from transcription start sites and potentially associated with transcriptional enhancers^3,4,7–9^. Interestingly, Myc interactions often do not overlap with conserved E-box motifs, particularly when Myc is overexpressed^5,9,10^. Such findings raise the possibility that Myc-driven tumorigenesis may extend beyond basic transcriptional control to a broader influence on other genomic processes.

DNA replication requires the stepwise assembly of protein complexes onto chromatin at future initiation sites. The origin recognition complex, Cdc6, and Cdt1 direct the loading of mini-chromosome maintenance (MCM2–7) hexamers onto DNA in a distributed manner throughout intergenic or transcriptionally inactive regions^11,12^. An excess of MCM hexamers beyond that required for a normal S-phase are loaded, the extra MCMs serving as dormant back-ups for recovering from replicative stress^13,14^. In late G1 in mammalian cells, Cdc45 and GINS (go-ichi-ni-san) are recruited to a subset of MCMs, forming replicative Cdc45-MCM-GINS (CMG) helicases^15–17^ that become active in replisomes at G1-S. The Cdc45 protein is present in cells at very low stoichiometric levels relative to MCMs, and its recruitment is rate-limiting for helicase formation and activation^18^. Regulating Cdc45 recruitment is therefore critical for helicase functionality and the initiation of DNA replication.

Myc also promotes tumorigenesis by causing genomic instability due to Myc’s ability to regulate DNA replication. Early studies demonstrated a role for Myc in embryonic S-phase whereby pools of maternally supplied Myc protein are taken up by nuclei preparing to initiate DNA replication^19^. DNA replication in early embryos does not rely on transcription, indicating that Myc provides an unknown non-transcriptional function. Loss of Myc impairs DNA replication, while excessive Myc levels produce over-activation of DNA replication origins^20,21^. Myc has been found in complexes with ORC and MCM subunits and colocalizes with some replication origins^20,22^. Origin hyperactivation under conditions of Myc overexpression involves a downstream effect of increased CMG activity, presumably from dormant MCM complexes, that is a requisite for DNA damage to occur^21^. While the loading of MCMs onto chromatin is not influenced by Myc^20^, Cdc45 and GINS recruitment is sensitive to an upstream Myc function^21^. Aside from indirect effects on Cdk activity^21^, no direct molecular mechanism for Myc in modulating Cdc45 or GINS recruitment or CMG activation has been shown. Similarly, whether Myc’s physical presence on chromatin influences CMG assembly and/or activation is not known.

Myc influences global chromatin modifications, in promoters as well as in promoter-distal intergenic regions^5^. The interaction of Myc with genomic sites correlates with acetylation of histones H3 and H4 indicative of euchromatin^10,23,24^. Myc interactions require preexisting active chromatin, but also further stabilize euchromatic changes^5,10,23^. Experimental loss of total Myc expression leads to global chromatin condensation and widespread heterochromatic histone modifications, while re-expression of Myc reverses these changes^23^. Such global chromatin condensation effects were shown to be an indirect result of the absence of Myc’s transcriptional control of the histone acetyltransferase GCN5 (KAT2A), rather than due to direct influences by Myc at specific sites. Myc also binds specific sites, such as promoters and enhancers, and recruits histone acetyltransferases (HATs) such as GCN5 and Tip60 (KAT5) to elicit gene regulation^25–27^, and Myc has been shown to be capable of causing nucleosomal rearrangements at promoters^28^. Beyond gene regulatory effects, it has been suggested that chromatin changes induced by Myc may further contribute to its oncogenicity, altering other genomic functions^5^. Whether DNA replication is included in such influences by Myc on chromatin remains unknown.

Here we demonstrate that targeting of Myc to genomic sites is associated with the assembly and activation of CMG helicases. Mechanistically, this derives from the ability of Myc to cause alterations in chromatin accessibility that facilitate, and are required for, the recruitment of Cdc45 and GINS to CMGs. Physiologic mediators of Myc biology, notably GCN5, Tip60, and TRRAP, are required, and the Myc-Box II domain critical for Myc-driven transformation must be intact for Myc to induce chromatin access, promote CMG assembly, and stimulate DNA replication. Importantly, Myc and GCN5 functions are required specifically in late G1 for Cdc45 assembly into CMGs and chromatin access at a chromosomal origin. Our findings support a model in which widely distributed genomic interactions by Myc influence chromatin structure in a manner that directly promotes the activation of CMG helicases at Myc-targeted regions, thereby explaining mechanistically how elevated Myc can promote the excessive DNA replication that contributes to oncogenic outcomes.

## Results

### Myc promotion of DNA replication and CMG activation requires Myc-box II

**Fig. 1:**
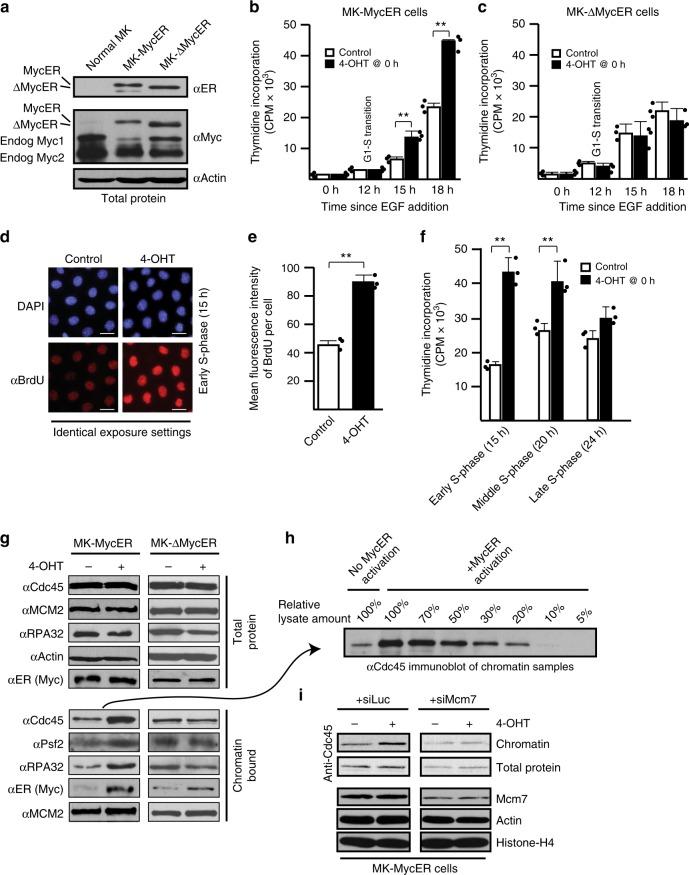
Myc promotes excessive CMG helicase assembly and activation that is dependent on Myc-box II function. **a** Immunoblots of MycER and ΔMycER protein expression in MK cells. **b**, **c** Tritiated-thymidine incorporation assays performed after MycER/ΔMycER activation in early G1 (0 h). Controls used carrier. **d** BrdU assay performed in early S (15 h) after MycER activation in early G1. Scale bars represent ~30 µm. **e** Software-based quantification of mean BrdU intensity/cell from **d**. **f** Synchronized MK cells with MycER activated in early G1, then analyzed by tritiated-thymidine incorporation assays at indicated times in S-phase. **g** Immunoblots of total or chromatin-bound proteins in early S (15 h) after MycER/ΔMycER activation in early G1. **h** Immunoblot of Cdc45 chromatin-bound samples from previous panel, with MycER-induced sample serially titrated to determine the increase in Cdc45 relative to uninduced. The 20% load is similar to uninduced, indicating ~5× increase. **i** Effects of partial siRNA knockdown of Mcm7 compared to siLuc control, ±MycER activation in early G1. Total and chromatin-bound Cdc45 was assessed by immunoblot in early S (15 h). Note that in each horizontal pair of blots, the data are from the same exposure of the same blot, but shown separated due to removal of irrelevant lanes for presentation purposes

Overexpression of Myc stimulates excessive DNA replication origin firing^21^. Consistent with this, mouse keratinocytes (Balb/MK) expressing regulatable MycER incorporate higher levels of radio-labeled thymidine into replicating DNA after MycER activation, but deletion of Myc-Box II (MBII; ΔMycER) renders Myc deficient for stimulating excessive DNA replication^29^. We re-created MK-MycER and MK-ΔMycER cells to allow investigation of other physiologic aspects of these Myc-driven events. Both ectopic Myc proteins express similarly (Fig. 1a). The MycER proteins are expressed at a level similar to endogenous Myc, and the sum of ectopic and endogenous Myc proteins is similar to the total Myc expression in normal MK cells. This indicates that MycER expression yields, at most, ~2-fold more Myc in total. Given that tumors are known to overexpress Myc up to 150-fold^30^, our MycER expression is well within physiologic levels. Activation of MycER in early G1 in synchronized MK cells stimulates excessive DNA replication during S-phase (Fig. 1b), while ΔMycER has no effect (Fig. 1c). Increased BrdU intensity per individual MycER-activated cell indicates that the excessive incorporation of DNA precursors results from extra DNA replication in each cell, rather than from more cells exiting quiescence (Fig. 1d, e). Flow sorting analysis also confirms that these MycER effects are not due to more cells exiting quiescence^29^. Notably, when MycER is activated in early G1, the stimulation of extra DNA replication is restricted to early-S and mid-S intervals, with late-S DNA replication activity being largely unaffected (Fig. 1f).

The excessive DNA replication origin usage induced by Myc is associated with more active CMG helicases^21^. The loading of rate-limiting Cdc45 relative to MCMs provides an indication of the number of assembled helicases^18^. In cell-free *Xenopus* replication assays, elevated Myc promotes the recruitment of additional Cdc45 to chromatin, and using ectopic Cdc45 as the readout, more Cdc45 is recruited during Myc overexpression in mammalian cells^20^. Activation of MycER in MK cells also causes an increase in the endogenous Cdc45:MCM ratio (Fig. 1g). MycER activation does not alter the amount of Mcm2 on chromatin, but does increase the association of Cdc45, Psf2, and RPA (single-stranded DNA (ssDNA)-binding protein), the latter a surrogate measure for functional helicase activity. Activation of ΔMycER does not affect these proteins. A titration assay shows that MycER activation raises Cdc45 levels on chromatin by ~5× (Fig. 1h), indicating a ~5-fold increase in CMGs, which aligns well with the predicted number of dormant MCMs available^14,18^. A partial knockdown of Mcm7, which reduces the levels of MCM hexamers that comprise the reserve/dormant pool^13^, prevents Cdc45 enrichment on chromatin after MycER activation, indicating that excessive Cdc45 is recruited to existing MCMs (Fig. 1i). Thus, activation of MycER with an intact MBII domain promotes excessive assembly of functional CMG helicases and higher levels of DNA replication.

### Myc induces chromatin unfolding at targeted genomic sites

**Fig. 2:**
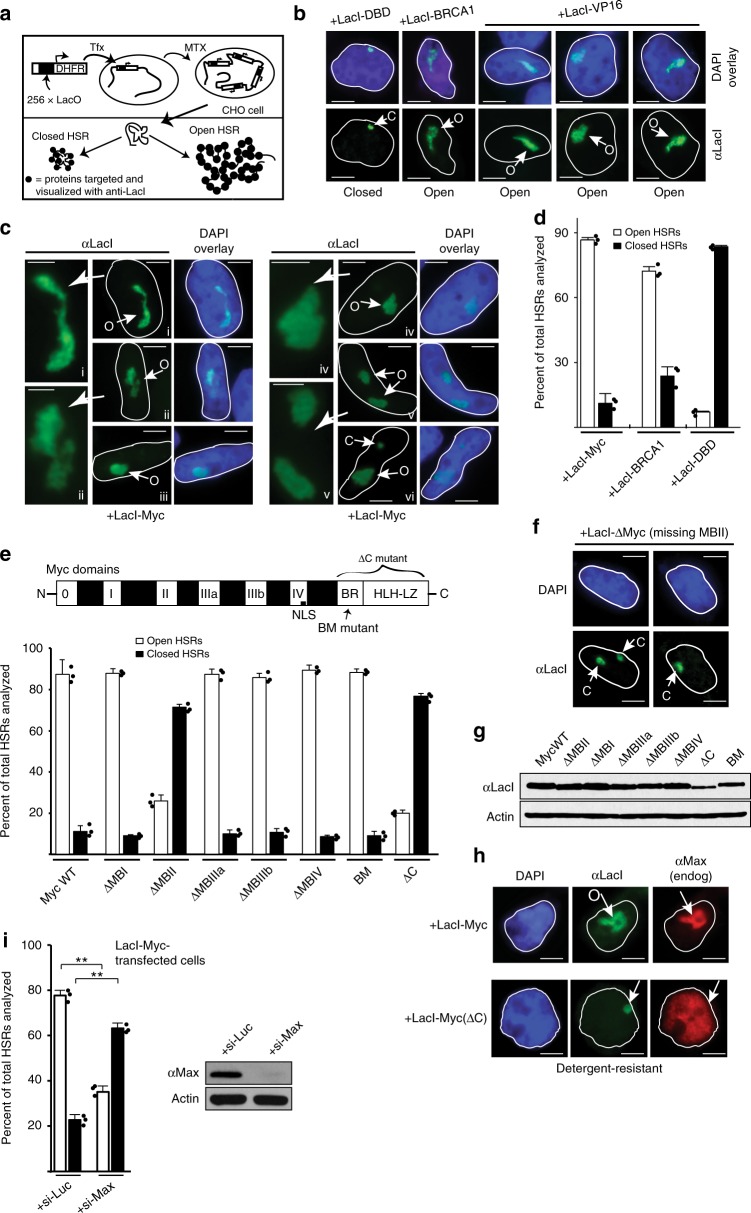
Targeting Myc to genomic sites induces higher-order chromatin decondensation. CHO (A03_1) cells were used for all chromatin unfolding assays. **a** Schematic of the chromatin remodeling system. **b** Examples of chromatin unfolding elicited by LacI-DBD, LacI-BRCA1 (6c-w variant), or LacI-VP16. HSRs visualized by immunofluorescence (IF) with anti-LacI (“O” for open HSRs, “C” for closed HSRs). Scale bars in all photomicrographs represent ~5 µm, unless noted otherwise. **c** Chromatin unfolding assays performed using LacI-Myc. Extended (i, ii), globular (iii–vi), or rare closed (vi) examples are shown. Panels i, ii, iv, and v are enlarged at left to show punctate appearance of open HSRs where LacI-Myc is concentrated within amplicons. Scale bars for enlargements represent ~2.5 µm. **d** Quantification of chromatin unfolding results for indicated proteins. **e** Quantification of chromatin unfolding results for mutant LacI-Myc proteins. **f** Chromatin unfolding assay examples for LacI-ΔMyc (MBII deletion). **g** Immunoblot showing similar expression for LacI-Myc proteins from **e**. **h** IF co-staining shows Max is recruited to HSRs unfolded by LacI-Myc, but not to closed HSRs that fail to unfold by LacI-ΔC-Myc targeting. **i** Max protein was reduced by siRNA for 48 h in A03_1 cells, verified in immunoblot. During the last 24 h, LacI-Myc was transfected and unfolding assays performed. Results were quantitated and graphed compared to siLuc control

We hypothesized that the ability of Myc to stimulate CMGs might derive from an influence by Myc on chromatin accessibility, facilitating helicase assembly and/or activation where Myc is bound and functioning in this regard. To test this concept, we employed an innovative chromatin remodeling assay system that tests the ability of targeted proteins to induce or inhibit higher-order chromatin unfolding^31–36^. This system utilizes a CHO-derived cell line with a 90 Mb homogeneous staining region (HSR) engineered through the stable insertion and methotrexate-based amplification of a vector containing Lac-operator (LacO) sites in the promoter of a dihydrofolate reductase gene (Fig. 2a). Cells can have one to three HSRs due to recombination events. This system allows for microscopic examination of chromatin structural changes that occur after targeting of LacI-fused proteins, and chromatin unfolding occurs as a result of physiologic mechanisms related to individually targeted proteins^33^.

The HSR natively adopts a condensed dot-like shape and is heterochromatic. Targeting the LacI DNA-binding domain (DBD) alone elicits no changes, showing a small closed HSR with anti-LacI immunofluorescent (IF) staining (Fig. 2b). However, targeting proteins such as VP16 or a BRCA1 variant, which recruit chromatin remodeling enzymes (e.g., HATs), causes a noticeable decondensation of the HSR (Fig. 2b). These unfolded HSRs appear as punctate globular or extended structures owing to the localization of targeted proteins to LacO sites within individual amplicons, and are several times larger than the native condensed HSR. These unfolded HSRs are called “open” or unfolded HSRs.

Similar to VP16 and BRCA1, and expected for a transcription factor that is associated with euchromatic histone marks, targeted LacI-Myc produces large-scale chromatin unfolding of the HSRs (Fig. 2c). The physical outcomes of Myc-opened HSRs are similar to VP16 and BRCA1, being extended (Fig. 2c, i and ii) or globular (Fig. 2c, iii and vi). In rare cases, Myc targeting does not produce a decondensation event, leaving the HSR closed (Fig. 2c, vi; note size differences between open and closed HSRs). Quantification of open versus closed HSR outcomes for Myc, BRCA1, and LacI-DBD was performed on multiple fields of transfected cells (Fig. 2d). As LacI-DBD is expressed at higher levels than other tested proteins^32^, its inability to unfold argues against protein crowding being involved. The LacI-Myc-induced HSR decondensation occurs in transfected cells that are in G1 or early S at the time of unfolding based on lack of co-staining for histone H1-phosphorylation^31^, which is evident in late-S and G2/M cells (Supplementary Figure 1A). Thus, Myc is capable of eliciting higher-order chromatin decondensation in this innovative system, and does so consistent with the timing of CMG assembly and activation in G1 or early S-phase. Below, we will also show that endogenous Myc is required for chromatin accessibility at a chromosomal replication origin known to bind Myc in vivo.

### Myc-driven chromatin decondensation requires Myc-box II and Max

We next determined the functional domains of Myc required for chromatin unfolding. A series of Myc deletion mutants were fused to LacI, and then compared quantitatively to wild-type LacI-Myc. Loss of MBI, MBIIIa, MBIIIb, and MBIV (but retaining the nuclear localization sequence) did not affect the ability of Myc to unfold chromatin (Fig. 2e). Similarly, loss of MB0, which is necessary for Myc-driven transactivation with MBI^37^, does not affect unfolding by Myc (Supplementary Figure 1B). However, loss of the MBII domain required for stimulating excessive DNA replication (Fig. 1c) renders Myc unable to induce chromatin unfolding (Fig. 2e, f; for the remainder of this study, ΔMyc mutants refer specifically to loss of MBII). Mutation of four residues in the basic DBD (BM-Myc) does not diminish chromatin unfolding, expected given the targeting of Myc with LacI, but loss of the HLH-LZ domain (ΔC-Myc) renders Myc deficient for decondensation (Fig. 2e). The LacI-Myc protein derivatives expressed similarly, with the exception of the ΔC-Myc mutant (Fig. 2g); however, conditions that produced similar expression of the latter relative to wt-Myc demonstrated that it remained incapable of unfolding chromatin (Supplementary Figure 1B).

The Myc-binding partner Max interacts with the HLH-LZ region^1^, and endogenous Max is recruited to LacI-Myc-unfolded HSRs (Fig. 2h), suggesting that Myc-Max interactions are involved in the mechanisms underlying Myc-induced chromatin decondensation. In support of this, Max is not recruited to (closed) HSRs targeted by the LacI-ΔC-Myc protein that cannot unfold chromatin (Fig. 2h), and reduction of endogenous Max levels by small interfering RNA (siRNA) renders LacI-Myc unable to efficiently unfold chromatin (Fig. 2i).

### Myc-driven chromatin unfolding is mediated by GCN5, Tip60, and TRRAP

**Fig. 3:**
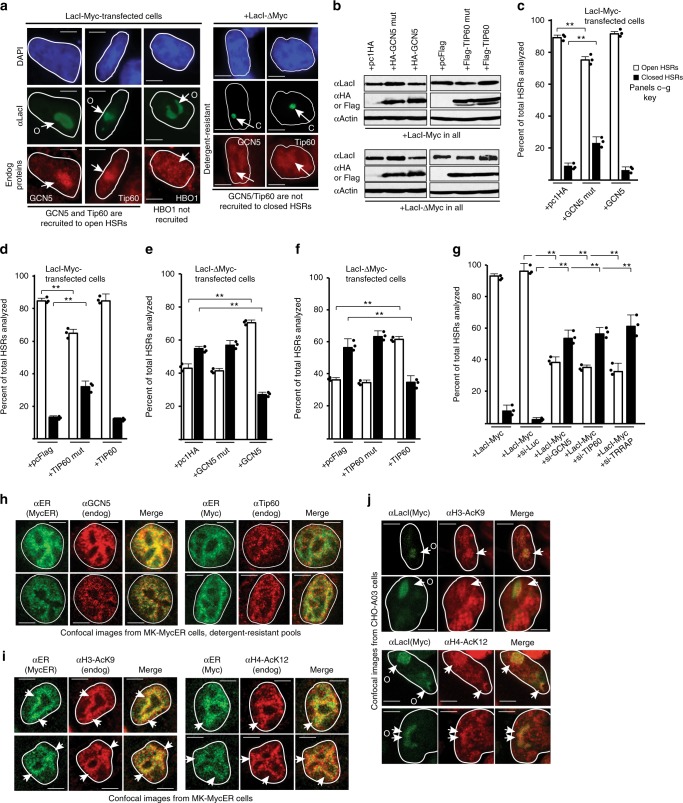
GCN5, Tip60, and TRRAP mediate Myc-driven chromatin unfolding. **a** Chromatin unfolding assays performed with LacI-Myc or LacI-ΔMyc and co-stained for recruitment of endogenous GCN5, Tip60, or HBO1. **b** HA-GCN5 or Flag-Tip60, or mutant versions, were transfected at a 2:1 ratio with LacI-Myc or LacI-ΔMyc, and expression assessed by immunoblotting. **c**–**f** Chromatin unfolding assays performed assessing effects of wild-type and mutant HATs on LacI-Myc or LacI-ΔMyc decondensing abilities. **g** Effects of partial siRNA-mediated knockdown of GCN5, Tip60, or TRRAP on the ability of LacI-Myc to unfold chromatin. **h** IF colocalization assays assessing overlap between MycER and GCN5 or Tip60 in MK cells. MycER was activated in early G1, and IF performed on cells in early S-phase during peak DNA replication stimulation by MycER (15 h). Arrows indicate points of reference for comparing certain features across panels. **i** Experiment performed as in **h**, but assessing overlap between MycER and acetylated H3 or H4. **j** Chromatin unfolding assays were examined for the presence of acetylated H3 or H4 at LacI-Myc-unfolded HSRs

Myc biology is known to involve the functions of GCN5 and Tip60 HATs, which interact with Myc in part through the MBII domain via TRRAP^5,23–27^. LacI-Myc targeting recruits GCN5 and Tip60 to open HSRs (Fig. 3a). Note that the bulk genomic IF signal for these enzymes is reduced due to photographic settings used to obtain non-overexposed images of the increased staining at the HSRs. The effect is specific for GCN5 and Tip60, as HBO1 is not recruited, consistent with studies indicating that HBO1 does not form complexes with Myc^25^. In contrast to that for LacI-Myc, LacI-ΔMyc does not recruit GCN5 or Tip60 to closed HSRs, with signal for both endogenous enzymes not enriched above bulk staining (Fig. 3a).

Next, wild-type or catalytically defective versions of GCN5 or Tip60 were co-expressed with LacI-Myc or LacI-ΔMyc (missing MBII), and chromatin decondensation efficiency was assessed under conditions where a mixture of endogenous and exogenous enzymes existed. Protein expression was similar for all factors tested (Fig. 3b). As a control, neither GCN5 nor Tip60 promote unfolding of the HSR targeted by LacI-DBD (Supplementary Figure 1C), indicating that the ectopic enzymes do not unfold the HSR on their own. While GCN5, Tip60, or an empty vector control did not alter the ability of LacI-Myc to promote higher-order chromatin unfolding, mutant GCN5 or mutant Tip60 caused a modest decrease in the efficiency of chromatin decondensation by Myc (Fig. 3c, d). Simultaneous co-expression of mutant GCN5 and mutant Tip60 caused a greater reduction in the ability of LacI-Myc to unfold chromatin, indicating that both enzymes seem to be involved during the unfolding process (Supplementary Figure 1D). Intriguingly, co-expression of wild-type GCN5 or Tip60 rescued the ability of LacI-ΔMyc to induce chromatin decondensation (Fig. 3e, f).

We next asked if a more noticeable effect could be observed when endogenous GCN5, Tip60, or the cofactor TRRAP was suppressed by siRNA-mediated methods (Supplementary Figure 1E, F). Partial knockdown of GCN5, Tip60, or TRRAP caused a suppression of LacI-Myc’s ability to unfold chromatin (Fig. 3g). Pharmacologic inhibition of GCN5 can be achieved using butyrolactone-3 (BL-3). Inhibition of endogenous GCN5 with BL-3 during the LacI-Myc transfection also reduced the efficiency of Myc-induced chromatin unfolding (Supplementary Figure 1G). These results demonstrate that the large-scale chromatin unfolding induced by LacI-Myc relies on mediators relevant to Myc biology (GCN5 and Tip60) and argue strongly that the decondensation is physiologically relevant to molecular mechanisms underlying Myc function.

Activation of physiologic levels of MycER was also strongly associated with GCN5 and Tip60 at genomic regions bound by MycER during early S-phase (Fig. 3h). Cells with differing levels of exposure were analyzed, and those with unique distributions of MycER are shown to illustrate that the overlaps of signal are indicative of a biological relationship, rather than due to random chance as might be presumed for evenly distributed focal patterns. We quantified the colocalization rates across several hundred cells in multiple fields (Supplementary Table 1). The colocalization rate between MycER and GCN5 or Tip60 was high, at 47 and 43% (Pearson *r*-coefficient values 0.78 and 0.72), respectively. In contrast, the colocalization rate between MycER and HBO1 was only 14% (*r* = 0.38).

Histone H3AcK9 and H4AcK12 modifications regulated by GCN5 and Tip60, respectively, were also associated with MycER-bound genomic regions at high levels (Fig. 3i and Supplementary Table 1; 42% or 41% colocalization rates, respectively), consistent with findings by others^25^. The same H3/H4 acetylations were also enriched at HSRs unfolded by LacI-Myc (Fig. 3j). Thus, GCN5 and Tip60 are recruited and required for Myc-targeted chromatin unfolding, and MycER and these enzymes co-exist on chromatin under physiologic conditions.

### Myc-induced chromatin unfolding does not influence MCM distribution

**Fig. 4:**
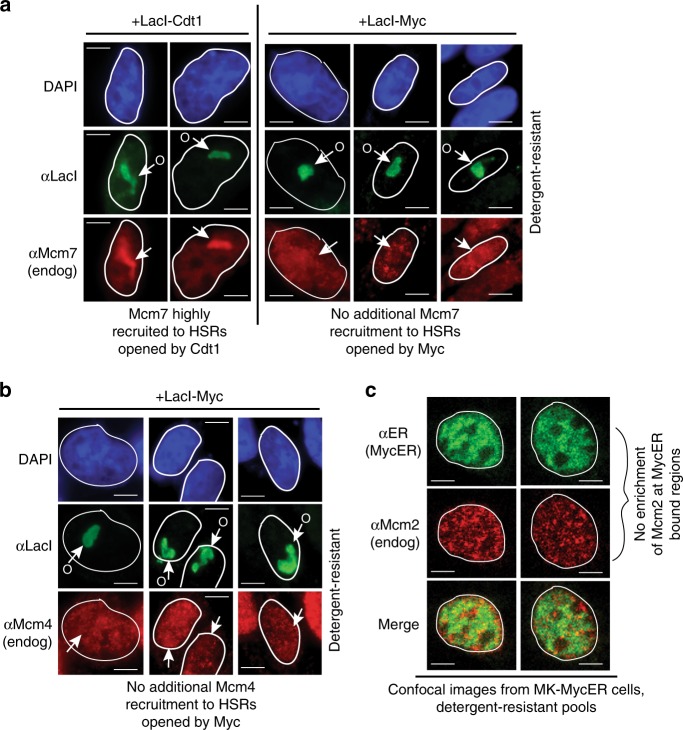
Myc-driven chromatin unfolding does not affect MCM distribution. **a** Chromatin unfolding assays performed with LacI-Cdt1 or LacI-Myc, and assessed for recruitment of Mcm7 to decondensed HSRs. **b** Chromatin unfolding assays assessed for recruitment of Mcm4 to HSRs decondensed by LacI-Myc. **c** IF colocalization assays assessing distribution between chromatin-bound MycER and Mcm2 in MK cells. MycER was activated in early G1, and IF performed on cells in early S-phase during peak DNA replication stimulation by MycER (15 h)

We next investigated the effects of Myc targeting on CMG dynamics. As a control, LacI-Cdt1 targeting increases recruitment of MCMs at unfolded HSRs due to the ability of Cdt1 to recruit HBO1 and load MCMs^32^. Mcm7 is noticeably enriched at HSRs unfolded by LacI-Cdt1 above normal global chromatin distribution patterns (Fig. 4a). However, targeting LacI-Myc does not cause enrichment of Mcm7 or Mcm4 to unfolded HSRs (Fig. 4a, b). Mcm4/7 staining is not absent from open HSRs, but rather is present at similar levels as bulk MCM staining throughout the genome. Thus, while Myc does not alter MCM distribution at open HSRs, LacI-Myc and MCMs co-reside in such regions. Under physiologic conditions, chromatin regions bound by activated MycER also do not show enrichment of Mcm2 (Fig. 4c). However, due to their punctate distribution, there is some overlap and spatial juxtapositioning between MycER and Mcm2, consistent with their colocalization rate being a moderate 26% (Supplementary Table 1; *r* = 0.52). These results indicate that interactions of Myc with the genome do not cause MCM enrichment (or re-distribution) at Myc-bound regions, and Myc/MCMs reside on chromatin in proximity to one another. These results are consistent with findings that Myc can exist in complexes with MCMs, but does not regulate MCM loading^20^.

### Chromatin unfolding by Myc regulates Cdc45 recruitment

**Fig. 5:**
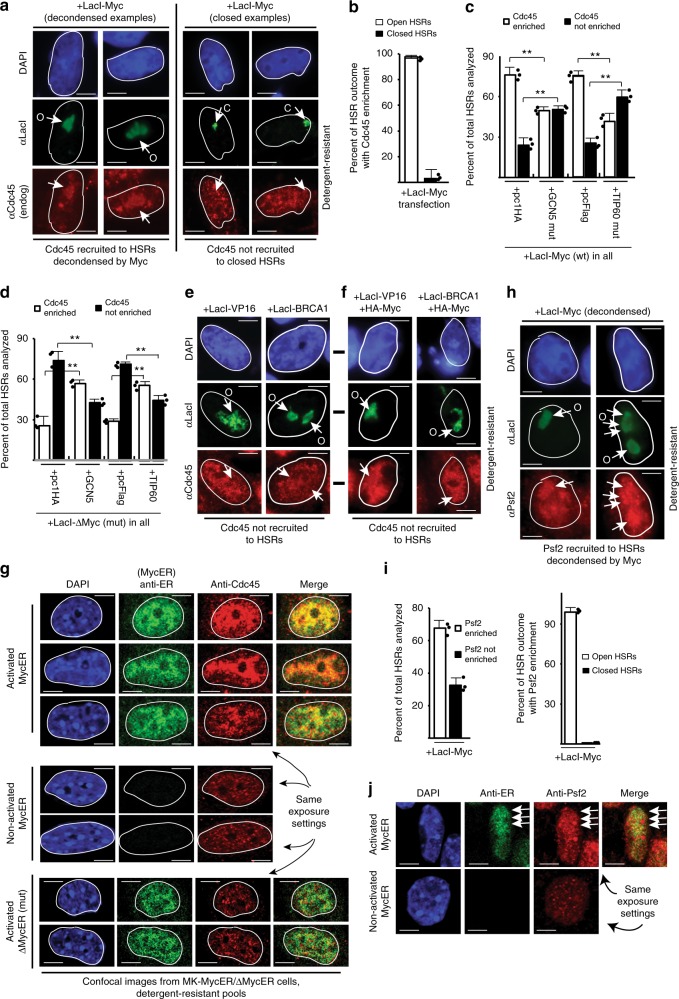
CMG helicase assembly is stimulated by, and dependent on, Myc-driven chromatin unfolding. **a** Chromatin unfolding assays performed with LacI-Myc and assessed for recruitment of Cdc45 to resultant decondensed (left) or non-decondensed (right) HSRs. **b** Quantification of Cdc45 recruitment to LacI-Myc-targeted HSRs from experiment in **a**. **c**, **d** The experiment used for immunoblotting in Fig. 3b was analyzed here for effects of GCN5 or Tip60, or mutant versions, on Cdc45 recruitment to HSRs targeted by LacI-Myc (**c**) or LacI-ΔMyc (**d**). **e** Chromatin unfolding assays were performed with LacI-VP16 or LacI-BRCA1(6c-w) and assessed for (lack of) Cdc45 recruitment to decondensed HSRs. **f** LacI-VP16 or LacI-BRCA1(6c-w) were co-expressed with HA-Myc, and chromatin unfolding assays were performed to assess (lack of) Cdc45 recruitment to decondensed HSRs. **g** IF colocalization assays in MK cells assessing distribution between endogenous Cdc45 and activated or non-activated MycER (top and middle panel sets), and versus activated ΔMycER (lower panel sets). MycER proteins were activated in early G1, and IF performed on cells in early S-phase (15 h). The same exposure settings were used for DAPI, anti-ER, and anti-Cdc45 comparisons. **h** Chromatin unfolding assays performed with LacI-Myc and assessed for recruitment of Psf2 to decondensed HSRs. **i** Quantitation of Psf2 recruitment to total HSRs targeted by LacI-Myc (left) and amount of Psf2 recruited to open or closed HSRs after LacI-Myc targeting (right). **j** IF colocalization assays assessing distribution between MycER and Psf2 in MK cells. MycER was activated in early G1, and IF performed on cells in early S-phase (15 h). Arrowheads indicate areas for comparison

Although Myc functions upstream of Cdc45 loading onto MCMs, the mechanisms by which Myc influences Cdc45 recruitment remain unclear^21^. We asked if targeting Myc to the HSRs, followed by chromatin decompaction, affected Cdc45 recruitment to resident MCMs. Figure 5a, b shows that Myc-driven decondensation promotes enrichment of Cdc45 at unfolded HSRs. Importantly, the few numbers of HSRs that were bound by LacI-Myc, but failed to unfold, did not recruit Cdc45. Thus, the binding of LacI-Myc to targeted HSRs alone, without decompaction, is not sufficient to recruit Cdc45. Because Myc-driven Cdc45 enrichment on chromatin is also MCM-dependent (Fig. 1h), Cdc45 is being recruited to resident MCMs within the HSRs following Myc-induced chromatin unfolding.

We next assessed if GCN5 and Tip60 mediated Cdc45 recruitment to Myc-unfolded HSRs. Mutant GCN5 or Tip60 co-expressed with LacI-Myc reduces the percentage of total HSRs that recruit Cdc45 (Fig. 5c). Conversely, co-expression of intact GCN5 or Tip60 reverses the inability of LacI-ΔMyc to recruit Cdc45 to HSRs (Fig. 5d). Thus, GCN5 and Tip60 overcome loss of MBII and their functions are required for Myc-induced chromatin unfolding and Cdc45 recruitment.

We showed in a prior report that Cdc45 targeting itself could unfold chromatin in this system via Cdk2 recruitment and H1-phosphorylation^31^. Since LacI-Myc was recruiting endogenous Cdc45, it was possible that Cdk2 was also involved in the LacI-Myc-induced unfolding. However, co-treatment with the Cdk2 inhibitor roscovitine did not affect the ability of LacI-Myc to unfold the HSRs (Supplementary Figure 1G), indicating that chromatin unfolding by LacI-Myc is not dependent on secondary recruitment of Cdk2.

Next, we determined if Cdc45 recruitment could be induced by other targeted proteins. HSRs unfolded by LacI-VP16 or LacI-BRCA1 did not recruit Cdc45 (Fig. 5e), indicating that Cdc45 recruitment was specific for Myc-driven decondensation. Cdc45 recruitment might also be due indirectly to LacI-Myc overexpression causing changes in the expression of a Myc-regulated protein(s) that itself causes Cdc45 recruitment to HSRs unfolded by any capable protein. To test this, we co-expressed hemagglutinin (HA)-tagged Myc with LacI-VP16 or LacI-BRCA1 and assessed Cdc45 recruitment. Cdc45 does not become enriched at VP16- or BRCA1-unfolded HSRs when HA-Myc is co-expressed (Fig. 5f and Supplementary Figure 2A, B). Under these conditions, HA-Myc was expressed at equal or greater levels than LacI-Myc (Supplementary Figure 2C). Thus, Myc specifically must be physically targeted to the HSRs, and induce chromatin unfolding, to promote Cdc45 recruitment.

We next examined the relationship between MycER activation, chromatin localization, and Cdc45 recruitment under physiologic conditions in early S-phase. In the absence of MycER activation, no ectopic Myc protein is stably bound to chromatin and Cdc45 genome-wide chromatin interactions are visible in an evenly distributed, punctate pattern that is generally uniform in intensity (Fig. 5g). Upon MycER activation, the Cdc45 chromatin interactions and IF intensity dramatically change, producing an elevation of Cdc45 recruited stably to chromatin in regions where MycER has the highest genomic interactions (Fig. 5g). A spatial overlap between MycER and Cdc45 patterns is clearly visible, with a high colocalization rate of 54% (Supplementary Table 1; *r* = 0.82). In contrast, activation of ΔMycER does not alter Cdc45 chromatin interactions relative to non-activated conditions, nor is there a discernible overlap in IF signals, with a low 15% colocalization rate (Fig. 5g and Supplementary Table 1; *r* = 0.38). We conclude that targeting Myc to chromatin under physiologic conditions and via tethering induces an excessive recruitment of Cdc45 to resident MCMs, and that local chromatin accessibility generated by targeted Myc/GCN5/Tip60 contributes mechanistically to this process.

### Myc-driven chromatin accessibility induces CMG assembly/activation

Similar to Cdc45, the GINS subunit Psf2 is recruited to the majority of Myc-unfolded HSRs (Fig. 5h, i). Under physiologic conditions that cause excessive DNA replication, Psf2 also stably associates with MycER-bound chromatin sites with a colocalization rate of 55% similar to Cdc45 (Fig. 5j and Supplementary Table 1; *r* = 0.72). Compared to non-activated MycER conditions, where Psf2 is evenly distributed in a punctate pattern similar to Cdc45, activation of MycER leads to an increased intensity of Psf2 signal at MycER-bound chromatin regions (Fig. 5j, arrows). The intensity differences are somewhat lower relative to that of Cdc45 (Fig. 5g), owing likely to the greater difficulty of using the anti-Psf2 antibody in our experiments.

**Fig. 6:**
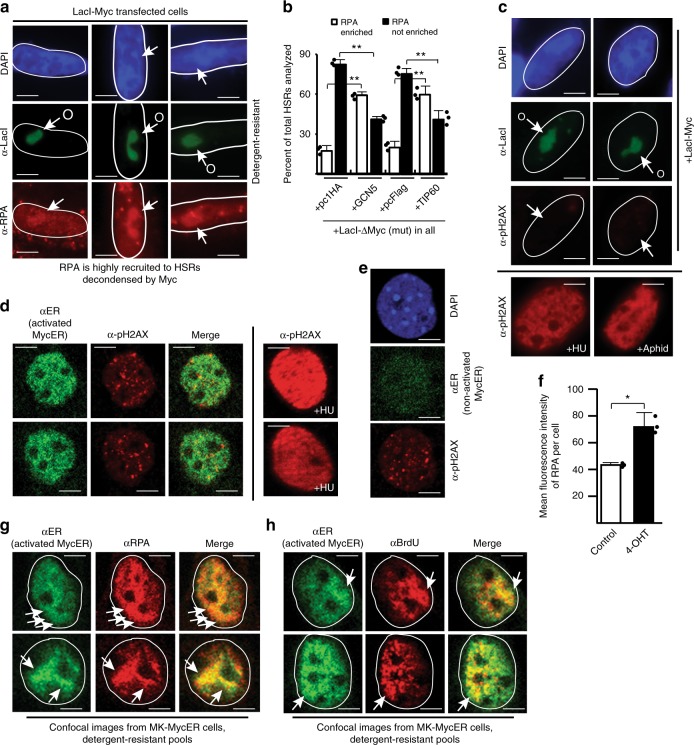
CMG helicase activation occurs at Myc-targeted genomic sites. **a** Chromatin unfolding assays performed with LacI-Myc and assessed for recruitment of RPA to decondensed HSRs. **b** The experiment used for immunoblotting in Fig. 3b was analyzed here for effects of GCN5 or Tip60 on RPA recruitment to HSRs targeted by LacI-ΔMyc. **c** Chromatin unfolding assays performed with LacI-Myc were assessed for (lack of) enrichment of pH2AX. Two cells at bottom are shown as positive controls for pH2AX elevation after hydroxyurea (HU) or aphidicolin treatment. **d** MK cells were assessed for (lack of) pH2AX enrichment at MycER-bound sites. Two positive control cells are shown at right for pH2AX increases after HU. **e** MK cell with non-activated MycER is shown as a comparison for pH2AX foci relative to activated MycER cells in **d**. **f** RPA staining performed in early S (15 h) after MycER activation in early G1. Software-based quantification of mean RPA intensity/cell from control and MycER-activated fields are shown. **g**, **h** IF colocalization assays assessing distribution between activated MycER and RPA or BrdU in MK cells. MycER was activated in early G1, and IF performed on cells in early S-phase at peak DNA replication stimulation after MycER activation (15 h). BrdU was pulsed for 30 min prior to fixation. Arrows denote areas for comparison. Exposures for RPA and BrdU signals were reduced to show detail and overlapping intensities of enrichment at regions with MycER

The recruitment of Cdc45 and GINS by Myc suggested that functional CMG helicases were forming. Helicase activity is measured by the recruitment of stably bound RPA to unwound ssDNA^38^. LacI-Myc targeting results in an enrichment of extraction-resistant RPA at unfolded HSRs (Fig. 6a). Whereas targeting of LacI-ΔMyc does not result in unfolding or RPA enrichment at HSRs, GCN5 and Tip60 co-expression with LacI-ΔMyc promotes RPA enrichment (Fig. 6b). Since Myc overexpression produces DNA damage with increases in global levels of phosphorylated histone H2AX (pH2AX)^21^, it was possible that LacI-Myc tethering was inducing DNA damage at sites of Myc interaction that promoted RPA recruitment as part of a repair process. However, pH2AX could not be detected at LacI-Myc-unfolded HSRs (Fig. 6c). We also found that pH2AX was not enriched at MycER-bound regions after activation (Fig. 6d). The small number of punctate foci for pH2AX present in MycER-activated nuclei are similar to that seen in non-activated MycER cells (compare Fig. 6d, e). These results indicate that neither MycER activation nor Myc tethering creates acute DNA damage at sites of Myc interaction.

Activation of MycER under physiologic conditions of excessive DNA replication results in an increase in the RPA signal intensity/cell relative to non-activated cells (Fig. 6f), similar to that for BrdU (Fig. 1d). Closer examination reveals that stably bound RPA is enriched at regions with high levels of MycER binding (Fig. 6g). Regions of highest MycER binding are also enriched for BrdU-labeled replication sites (Fig. 6h). Cells with atypical (non-even) MycER patterns are shown with reduced exposures for RPA/BrdU to illustrate that the spatial overlaps of these events with MycER derive from a biological relationship, versus random chance. The colocalization rate between RPA and BrdU versus MycER is 54 and 55%, respectively (Supplementary Table 1; *r* = 0.90–0.91). Notably, this colocalization rate exists for cells in early S-phase; cells in middle or later S-phase have lower colocalization rates of 23 and 22%, respectively *r* = 0.59–0.60). The colocalization between RPA/BrdU and MycER is consistent with a report showing that endogenous Myc colocalizes with replication sites^20^. These results support a model in which targeting Myc to genomic regions induces localized hyperactivation of functional CMG helicases, and that Myc-driven chromatin accessibility is mechanistically involved in this process.

### Myc binds Cdc45, and Myc/MBII are required for Cdc45 loading into CMGs

**Fig. 7:**
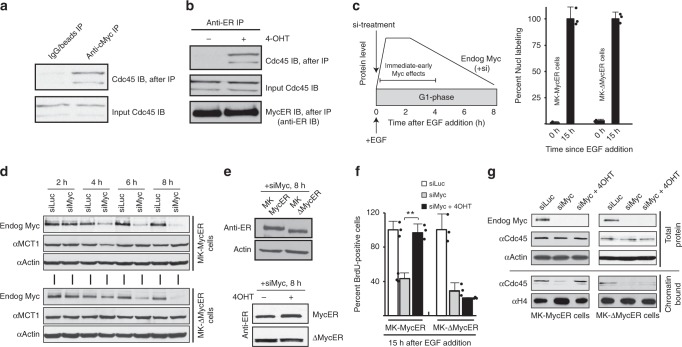
Myc interacts with Cdc45, and Myc/MBII are required in late G1 for Cdc45 recruitment to CMGs. **a**, **b** Synchronized MK-MycER lysates from late G1 (11 h) subjected to immunoprecipitation (IP), then immunoblotting (IB). Endog Myc:Cdc45 is in **a**, and MycER:Cdc45 ± ectopic protein activation is in **b**. Anti-Cdc45 used here in IB probings, which recognizes two bands, was a mouse monoclonal antibody to prevent interference from heavy chain reactivity. Similar results were obtained in separate biological replicates. **c** Experimental design for **d**–**g**. Verification of EGF-synchronization of MK/MycER or MK/ΔMycER cells by BrdU analysis. **d** MK cells transfected at start of G1 with siMyc or siLuciferase, then analyzed by immunoblot at times shown. **e** Similar amounts of MycER and ΔMycER proteins in late G1 after siMyc exposure (top), and similar levels of both proteins ± inducer (bottom). **f** After treatment with siLuc or siMyc, ±MycER or ΔMycER activation in early G1, the ability of MK cells to enter S-phase was quantified using BrdU (15 h after release). **g** For the experiment in **f**, immunoblot of total or chromatin-bound samples at time of BrdU analysis

Myc can form complexes with MCMs, at least indirectly^20^, and our data suggested it might also form complexes with the subset of MCMs that become CMGs. To test this, we determined if Myc could form complexes with Cdc45. Immunoprecipitation (IP) with anti-Myc followed by immunoblotting with anti-Cdc45 demonstrated that endogenous Myc does form complexes with Cdc45 in late G1 (Fig. 7a). Similarly, MycER forms complexes with Cdc45 in late G1, but only when activated (Fig. 7b).

We next determined if Myc (and the MBII domain) were required specifically in late G1, at the time Cdc45 interacts with Myc and loads onto chromatin. Cdc45 recruitment to CMGs begins at ~8 h in the 12-h G1 interval in synchronized MK cells^16^. Myc is required for early-G1 transcription of genes important for G1 progression^1^. We did not want to disrupt the ability of Myc to regulate such transcription, which might indirectly block Cdc45 loading and other late-G1 events. We devised an experiment (Fig. 7c) in which synchronized MK cells released into G1 were immediately transfected with siRNA targeting murine Myc or Luciferase (Luc). Due to Myc’s lability, this would confer a delayed loss of endogenous Myc, allowing it to be present in early G1, but absent in late G1. MK-MycER or MK-ΔMycER cells were used to allow for concurrent activation of ectopic human Myc (less sensitive to murine siRNA), and the results in Fig. 7c show that we efficiently synchronized both cell types.

As predicted, endogenous Myc was present in the early-G1 interval and was lost by late G1 (6–8 h) in both cell types exposed to siMyc (Fig. 7d). Under such conditions, early-G1 gene regulatory events elicited by Myc were intact, as shown by the unaltered expression of the Myc target MCT1 (Fig. 7d)^39^. MycER and ΔMycER proteins were both present in late G1 at similar levels, and activation with inducer did not alter their levels (Fig. 7e). Delayed loss of Myc specifically in the late-G1 interval resulted in a block to S-phase entry (Fig. 7f), consistent with Myc function being required during most of G1^1,40^. Activation of MycER, but not ΔMycER, during siMyc treatment rescued the ability of cells to enter S-phase. Suppression of Myc in late G1 also inhibited Cdc45 loading onto chromatin (Fig. 7g), which was rescued by activation of MycER. However, activation of MBII-deficient ΔMycER did not rescue Cdc45 loading. Thus, a function of Myc derived from MBII is required specifically during late G1 for Cdc45 to bind to MCMs on chromatin and allow cells to enter S-phase with functional CMGs.

### Myc and GCN5 are required for accessibility at a chromosomal origin

**Fig. 8:**
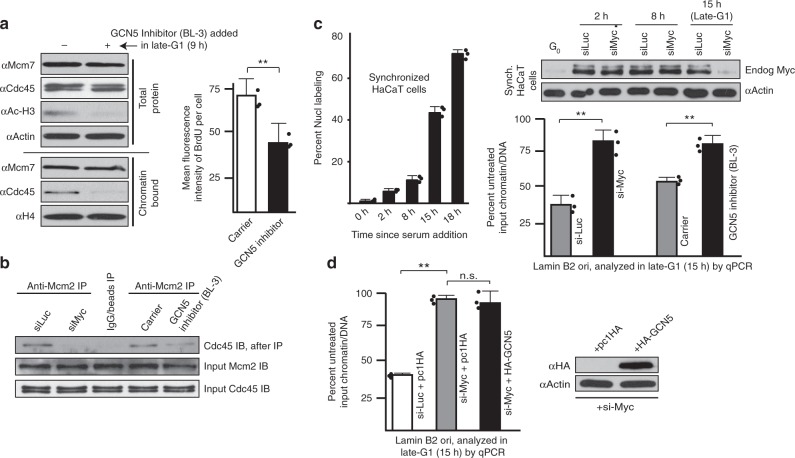
GCN5 regulates Cdc45 recruitment to CMGs, and is required with Myc for chromatin access at a chromosomal replication origin. **a** Synchronized MK cells treated with GCN5 inhibitor butyrolactone-3 (BL-3) in late G1 (9 h), and total and chromatin-bound protein lysates from early S-phase (15 h) analyzed by immunoblotting (left). BrdU incorporation/cell was assessed at the same time as protein collection (right). **b** Synchronized MK cells transfected with siMyc at the start of G1 or treated with BL-3 in late G1 (9 h), and lysates collected at 12 h and subjected to IP, then IB. **c** HaCaT cells synchronized by serum deprivation 24 h, verified by BrdU assays. HaCaT were transfected with siMyc at the start of G1 or treated with BL-3 in late G1 (12 h), and at 15 h chromatin was isolated and used as a substrate for origin accessibility assays. Immunoblot verifies endogenous Myc protein loss by late G1. Results are means from three separate qPCR runs, each in triplicate. Results are representative of two biological replicates with similar results. **d** An experiment was performed as in **c**, but HA-GCN5 or empty vector control was expressed during siRNA-mediated loss of Myc. Ectopic protein expression at time of qPCR sampling verified in immunoblot

We next determined if GCN5 function was required specifically in late G1 for Cdc45 recruitment to chromatin and S-phase entry. Synchronized MK cells were exposed to BL-3 in late G1 to inhibit endogenous GCN5. Reduction of acetylated H3 verified that the BL-3 drug was effective at suppressing GCN5 activity in cells (Fig. 8a). GCN5 inhibition in late G1 resulted in reduction of Cdc45 on chromatin and a reduced ability of cells to replicate DNA (Fig. 8a). We next asked if, consistent with a lack of Cdc45 on chromatin, the late-G1 loss of Myc or inhibition of GCN5 prevented Cdc45 from loading into CMGs. Cdc45 is known to interact with Mcm2 in the CMG complex, and siMyc or GCN5 inhibition blocks such interactions (Fig. 8b). Therefore, Myc, MBII, and GCN5 functions are required in late G1 for Cdc45 assembly into CMG helicases.

The human Lamin B2 DNA replication origin has been shown to contain an E-box bound by Myc in middle-late G1, with loss of Myc interactions correlating with reduced origin activity^22^. We asked whether loss of Myc in late G1 or acute GCN5 inhibition affected chromatin accessibility at this origin. We described an innovative approach^32^ in which quantitative PCR (qPCR) is performed on the Lamin B2 origin after chromatin samples are pre-exposed to DNase-I. If chromatin at the origin is accessible, then DNase-I can enter and slightly digest the substrate DNA, rendering more cycles necessary to amplify relative to untreated DNA (or chromatin-closed DNA). We synchronized human HaCaT cells (to analyze human origin) and treated with siMyc in early G1, or siControl, to reduce Myc protein levels by late G1 (Fig. 8c; synchronization verified in graph, with late G1 or G1/S at ~15 h). Parallel samples were treated with BL-3 in late G1 to acutely inhibit endogenous GCN5. Relative to siControl, late-G1 loss of Myc or inhibition of GCN5 renders the Lamin B2 origin less accessible to DNase-I digestion (more PCR product), indicating that the chromatin at the Lamin B2 origin is more compact with Myc absent or GCN5 inhibited. Figure 8d shows that GCN5 overexpression cannot rescue Myc loss, indicating that GCN5’s role in origin accessibility is dependent on Myc’s presence. Thus, the functions of Myc and GCN5 create chromatin accessibility at a chromosomal replication origin.

## Discussion

Myc is capable of influencing global chromatin architecture and its genomic interactions are associated with euchromatic marks^5,23,41^. Histone H3 and H4 modifications are not only prerequisites for Myc binding but are also further modified following Myc binding^9,10,23,24^. Using an engineered tethering system in yeast, Myc caused remodeling of nucleosome positioning in a promoter that induced transcriptional activity^28^. Consistent with these findings, we used a similar approach to show that targeting of Myc elicits HAT-dependent large-scale changes to higher-order chromatin structure at Myc-bound regions.

Cdc45 and GINS recruitment to MCMs is downstream of Myc function, and one indirect mechanism is a Myc-dependent influence on CDK activity^21^. Our work demonstrates that Myc also has a direct, non-transcriptional role in regulating CMG assembly and activation that involves a Myc-driven creation of higher-order chromatin accessibility. The experimental manipulation of Myc’s localization to genomic sites coincides with activation of CMGs at Myc-targeted regions. Mechanistically, Cdc45 and GINS recruitment to MCMs is regulated by the genomic targeting of Myc and consequent decompaction of chromatin that yields an accessible state for localized CMG assembly at preexisting MCMs. Such results are consistent with Myc being found in complexes containing CMG subunits [ref. ^20^, and results herein]. Myc’s ability to induce large-scale chromatin changes that stimulate CMG activity agrees with evidence that epigenetic factors drive the initiation of DNA replication in higher eukaryotes^42^.

Consistent with our ectopic approaches, we show that endogenous Myc and GCN5 functions are required for chromatin accessibility in late G1 at the Lamin B2 chromosomal replication origin, which is known to bind Myc via E-box interactions during such interval^22^. Loss of Myc diminishes activity of this origin^22^, in agreement with our results demonstrating that Myc/GCN5 are needed for chromatin access and CMG assembly. Although this previous study showed that Mcm4 recruitment to the Lamin origin correlated with Myc’s presence, our results and those by others^20^ indicate that Myc does not itself influence MCM assembly. In addition, HBO1 recruitment to the Lamin origin correlated with Myc availability^22^, but our findings and those of others^25^ indicate that Myc does not recruit HBO1 as part of its chromatin access function. One likely explanation for these differences is that the Lamin origin also recruits Cdt1, which is known to modulate MCM assembly via HBO1 recruitment^32^. Thus, multiple origin-regulatory events involving chromatin access, MCM assembly, and CMG activation are occurring that are collectively required for DNA replication to initiate. Important to appreciate is the fact that Myc overexpression does not influence all replication origins (MCM sites), since we showed that late-S replication dynamics are unaffected by Myc. Therefore, while Myc can clearly regulate chromatin access and CMG assembly at some origins, all potential replication origins are not controlled by Myc.

The unfolding of chromatin and activation of CMGs at Myc-targeted sites are dependent on cofactors that function in Myc biology. GCN5 and Tip60 are required, and the acetylation of H3 ad H4 is evident at Myc-targeted and unfolded sites^9,25^. TRRAP is also required for Myc-induced unfolding, providing an explanation for why co-expression of GCN5 and Tip60 overcomes loss of Myc-Box II^25–27^. TRRAP and these HATs form complexes via multivalent contacts on Myc, one site being MBII and other site(s) being in the vicinity of MBI and MB0^26,37^. Thus, overexpressing the HATs may overcome loss of MBII by facilitating weakened biochemical interactions with Myc, promoting sufficient chromatin unfolding. It is also interesting that VP16 can recruit GCN5 and TRRAP, unfold chromatin^43^, yet not induce CMG assembly (data herein). This indicates that CMG assembly is specifically influenced by the targeting of specific macromolecular complexes containing Myc, GCN5, Tip60, and TRRAP, rather than by the enzymes themselves. It is thus possible that interactions of Myc itself with subunits of the CMG^20^ also contribute to helicase assembly beyond simply needing chromatin access.

The fact that the MBII domain is required for Myc’s ability to stimulate DNA replication, unfold chromatin, and assemble active CMGs has important implications for carcinogenesis. The MBII domain is required for transformation by Myc, and although MBII is not strictly required for transactivation by Myc when assessed by standard transactivation assays^28,37,44–46^, it is required in the normal chromatinized context of genes for creating chromatin access as a precursor step in transactivation^46^. Our results here indicate that this function of Myc in chromatin modulation also contributes to stimulation of the DNA replication machinery. Myc is known to interact not only with promoters but also with a vast number of sites throughout the genome, including intergenic regions where MCMs reside in preparation for conversion to CMGs^3,4,7–9^. Such widespread genomic interactions are likely enhanced when Myc is overexpressed in cancer^5,9,10^. We contend that Myc’s interactions as such allow complexing with MCMs^20^ and local chromatin changes that facilitate CMG assembly similarly to how Myc controls promoters. In support, we show that Myc must be physically present specifically in late G1, with an intact MBII domain, to allow Cdc45 recruitment to MCMs. Notably, this late-G1 interval no longer requires de novo transcription for mammalian cells to transit G1/S^47,48^, yet Myc is nonetheless required to provide a function for HAT recruitment. Altogether, our findings indicate that Myc’s ability to directly regulate the replication apparatus via MBII-dependent chromatin accessibility changes likely plays an important and physiologically relevant role in Myc-driven tumorigenesis.

## Methods

### Cell culture, synchronizations, and inhibitors

Mouse keratinocytes (Balb/MK) were maintained in low-calcium minimum essential medium supplemented with 8% dialyzed fetal bovine serum (Hyclone) and 4 ng/ml epidermal growth factor (Invitrogen). The CHO cell line used for chromatin unfolding assays (clone A03_1) was maintained in Dulbecco’s modified Eagle’s medium (DMEM) supplemented with 10% Fetal Clone-2 (Hyclone) and 0.3 μM methotrexate. Transfections lasted 24 h with TransIT-LT1 (Mirus). MK lines expressing MycER or ΔMycER were created using retroviral methods, maintained in phenol red-free medium with charcoal-stripped serum, and synchronized by epidermal growth factor deprivation for 3.5 days^49^. MycER activation utilized 2 μM 4-hydroxytamoxifen (Sigma) in methanol carrier. HaCaT cells used for origin accessibility assays were maintained in DMEM supplemented with 10% fetal bovine serum (Omega Scientific), and synchronized by serum deprivation for 24 h. Verification of effective synchronizations was done using either BrdU-nuclear labeling/IF analysis or tritiated-thymidine scintillation counting assays, as described below. Such results are shown in several figures. All siRNA reagents were from Dharmacon and used at 100 nM. BL-3 (Abcam) was used at 100–200 µM in ethanol carrier. Roscovitine (EMD-Millipore) was used at 40 µM.

### Plasmids

pMV7-MycER/ΔMycER plasmids were described^49^. LacI-VP16 was provided by Dr. Andrew Belmont (University of Illinois). LacI-HsCdt1, LacI-BRCA1 (6c-w mutant), and all human LacI-Myc derivatives (c-Myc) were expressed in pRcLac^32^. Myc mutants were as follows (amino-acid deletions noted): MB0 (Δ10–32); MBI (Δ44–63); MBII (called ΔMyc; Δ106–143); MBIIIa (Δ188–199); MBIIIb (Δ259–270); MBIV (Δ304–319, NLS residues 320–328 intact); Myc-BM (residues 364–367 mutated from RQRR to ADAA); and Myc-ΔC (deleted C terminus, residues 353–439). The cDNA sequences within pIRES-GCN5 (murine WT; Kat2A) and pIRES-GCN5 (GYG-AYA mutant; residues 589–591) were subcloned into pcDNA3-HA. pcDNA3-Flag-TIP60 (human WT; Kat5), pcDNA3-Flag-TIP60 (GQE mutant; Q377E and G380E), and both pIRES plasmids were provided by Dr. Edward Seto (George Washington University).

### Antibodies

Antibodies are rabbit polyclonal or mouse monoclonal (mAb), unless stated. Working dilutions (IB, immunoblot and IF), catalog numbers, and clone/lot numbers for each antibody are indicated. From Santa Cruz Biotech: anti-Psf2 (1:200 IB, 1:50 IF; sc-98556, lotB0409), mAb anti-Cdc45 (1:150 IB after co-IP; sc-55569, cloneG12, lotB0317), anti-HBO1 (1:50; sc-25379, lot1813), anti-Tip60 (1:50 IF; sc-25378), mAb anti-Tip60 (1:200 IB; sc-166323, cloneC7, lotH2316), mAb anti-ER (1:200 IF and IB; sc-8002, cloneF10, lotJ0716), mAb anti-Mcm7 (1:1000; sc-9966, clone141.2, lotI0416), mAb anti-H3AcK9/14 (1:500 IB; sc-518011, cloneD4, lotJ3117), and mAb anti-MCT1(1:1000; sc-365501). From Sigma: mAb anti-Actin (1:5000; A5316, cloneAC74) and mAb anti-Flag (1:5000; F3165, cloneM2). From Cell Signaling: rat monoclonal anti-RPA32 (1:50; cat2208, clone4E4, lot2), anti-Myc (1:20 IP; cat9402, lot11), rabbit monoclonal anti-ER (1:20 IP; cat8644, cloneD8H8, lot4), rabbit monoclonal anti-GCN5 (1:500; cat3305, cloneC26A10, lot4), anti-TRRAP (1:500; cat3966, lot2), rabbit monoclonal anti-H3AcK9 (1:500; cat9649, cloneC5B1, lot11), rabbit monoclonal anti-H4AcK12 (1:500; cat13944, cloneD2W60, lot1), mAb anti-H4 (1:500; cat2935, cloneL64C1, lot6), and rabbit monoclonal anti-pH2AX (1:500; cat9718, clone20E3, lot10). From Millipore: anti-H1P (1:200; cat06597, lot2892887) and mAb anti-LacI (1:20,000; cat05503, clone9A5). From Stratagene: rabbit polyclonal anti-LacI (1:20,000; discontinued, but sample available upon request). From Covance: mAb anti-HA (1:500; MMS-101R, clone16B12, lot14831802). From Life Technologies: mAb anti-BrdU (1:50; B35132, cloneM0BU1, lot1626608). Anti-Myc (1:3000 IB) was provided by Dr. Steve Hann [Vanderbilt University]; chicken polyclonal anti-Cdc45, anti-Mcm2, and anti-Mcm4 (all at 1:1000; anti-Mcm2 1:20 for IP) were generated by our group and validated as described^18^.

### Immunoblotting and IPs

For immunoblotting with standard ECL and PAGE gel/transfer methods, lysates from equal cell numbers were separated into Triton X-100-soluble or -resistant (chromatin-bound) fractions as described^18^, and compared to whole-cell lysates. IPs were done at 4 °C on cells lysed in TNN (50 mM Tris-HCl, pH 8; 250 mM NaCl, and 0.1% Nonidet P-40). Primary antibodies were rocked with lysates for 1–2 h, then 1 h with anti-rabbit agarose beads blocked with 1% bovine serum albumin (Sigma), followed by 4 × 15 min (rocking) bead washes in excess TNN. All original IBs used to compose panels within each figure are shown in Supplementary Figures 3–8.

### Chromatin unfolding assays and immunofluorescence

Standard IF procedures and chromatin unfolding assays in A03_1 and MK cells were performed as described previously^32^. Note that for all chromatin unfolding and IF assays, experiments were performed a minimal of two times with independent biological replicates, with similar outcomes. To derive graphed averages (±1 s.d.), 50–60 A03_1 cells with a visible HSR (open or closed) were scored in three independent assessments across fields. For detecting chromatin-bound proteins in IF (for CHO and MK cells), prior to fixation, cells were pre-extracted for 10 min with cold extraction buffer (0.1% Triton X-100, phosphate-buffered saline, pH 7.4, 5 mM MgCl_2_, 0.1 mM EDTA, pH 8). Fluorochromes used were Alexa-594 (red) and Alexa-488 (green) conjugated to secondary antibodies (Jackson ImmunoResearch, Inc.). Images were obtained using a Zeiss Axio-observer Z1 upright widefield ultraviolet fluorescent microscope with high-resolution charge-coupled device camera, or a Leica SP5 AOBS inverted laser-scanning confocal microscope. A ×63/1.4 numerical aperture Plan Apochromat oil-immersion objective was used for image acquisition. Scale bars in all figures represent ~5 µm, unless noted otherwise. Colocalization rate analyses were performed using Leica Application Suite X software, and intensity measurements using Definiens Tissue Studio v4.2 software. All images were analyzed, sized, and assembled into figures using Adobe Photoshop and Illustrator software. Any gamma adjustments were performed evenly across entire images.

### Origin accessibility assay

Chromatin was isolated in a buffer containing 10 mM Tris-HCl (pH 7.5), 5 mM MgCl_2,_ 1 mM CaCl_2_, 10 mM KCl, 300 mM sucrose, and 0.1% Triton X-100 for 5 min on ice, then washed and resuspended with the same buffer lacking detergent. One-third of the chromatin from a 10 cm plate of cells was digested with DNase I (Promega) at 3 U/100 µL for 7 min at room temperature. Another third was treated identically, but without DNase I (untreated control used for normalization). Reactions were stopped by addition of 10 mM EDTA/2 mM EGTA and incubated at 65 °C for 10 min. DNA was lightly sonicated, then treated with 50 µg/mL RNase (Sigma) for 30 min at 37 °C and 250 µg/mL Proteinase K (Sigma) overnight at 42 °C. DNA was purified using phenol/chloroform extractions and analyzed using TaqMan-based qPCR (primer sequences available upon request). qPCR results were analyzed according to the formula 100/2^Ct(DNase I)−Ct(no DNase I)^. Using this method, measurements were normalized to input DNA (no DNase I treatment).

### DNA replication assays, synchronization verification, and statistics

Synchronization was verified in all noted experiments using one of two methods that measured replicating DNA (or lack thereof). DNA replication was quantitatively measured in MK cells by pulsing wells at indicated times with 3 μCi/mL of [^3^H]-thymidine for 30 min prior to fixation^29^. Trichloroacetic acid-precipitable material was analyzed by scintillation testing. Replicating DNA was also labeled with 15 μM BrdU for 30 min, and fixed and analyzed by IF for nuclear labeling percentages (for MK and HaCaT cell experiments). For statistics in replication assays and chromatin unfolding experiments (all performed in triplicate and averaged ±1 s.d.), two-tailed *t* tests were performed throughout, with significance asterisk values indicated as **P ≤ 0.01 or *P ≤ 0.05.

### Reporting summary

Further information on experimental design is available in the Nature Research Reporting Summary linked to this article.

## Supplementary information

Reporting Summary

Supplementary Information

## Data Availability

All relevant data within the manuscript are available for review or discussion by interested parties. Original immunoblots are included for all panels as Supplementary Information. If necessary, we can facilitate experiments planned by other parties that are related to the results of our study. There are no large datasets and accession codes that have contributed to the information in this paper.
