# Supplementary material for: Myc-driven chromatin accessibility regulates Cdc45 assembly into CMG helicases
Source: Commun Biol. 2019 Mar 22;2:110. doi: 10.1038/s42003-019-0353-2 (PMC6430796; doi:10.1038/s42003-019-0353-2)
Supplement: Supplementary file 1 — Reporting Summary [file 42003_2019_353_MOESM1_ESM.pdf]

nature research

Corresponding author(s): Mark G. Alexandrow

Reporting Summary

Nature Research wishes to improve the reproducibility of the work that we publish. This form provides structure for consistency and transparency in reporting. For further information on Nature Research policies, see Authors & Referees and the Editorial Policy Checklist.

Statistical parameters

When statistical analyses are reported, confirm that the following items are present in the relevant location (e.g. figure legend, table legend, main text, or Methods section).

n/a	Confirmed	
☐	☒	The exact sample size (*n*) for each experimental group/condition, given as a discrete number and unit of measurement
☐	☒	An indication of whether measurements were taken from distinct samples or whether the same sample was measured repeatedly
☐	☒	The statistical test(s) used AND whether they are one- or two-sided<br>*Only common tests should be described solely by name; describe more complex techniques in the Methods section.*
☒	☐	A description of all covariates tested
☒	☐	A description of any assumptions or corrections, such as tests of normality and adjustment for multiple comparisons
☒	☐	A full description of the statistics including central tendency (e.g. means) or other basic estimates (e.g. regression coefficient) AND variation (e.g. standard deviation) or associated estimates of uncertainty (e.g. confidence intervals)
☐	☒	For null hypothesis testing, the test statistic (e.g. *F*, *t*, *r*) with confidence intervals, effect sizes, degrees of freedom and *P* value noted<br>*Give P values as exact values whenever suitable.*
☒	☐	For Bayesian analysis, information on the choice of priors and Markov chain Monte Carlo settings
☒	☐	For hierarchical and complex designs, identification of the appropriate level for tests and full reporting of outcomes
☐	☒	Estimates of effect sizes (e.g. Cohen's *d*, Pearson's *r*), indicating how they were calculated
☐	☒	Clearly defined error bars<br>*State explicitly what error bars represent (e.g. SD, SE, CI)*

Our web collection on statistics for biologists may be useful.

Software and code

Policy information about availability of computer code

Data collection	No custom software was used in this report
Data analysis	No custom software was used in this report

For manuscripts utilizing custom algorithms or software that are central to the research but not yet described in published literature, software must be made available to editors/reviewers upon request. We strongly encourage code deposition in a community repository (e.g. GitHub). See the Nature Research guidelines for submitting code & software for further information.

Data

Policy information about availability of data

All manuscripts must include a data availability statement. This statement should provide the following information, where applicable:

- Accession codes, unique identifiers, or web links for publicly available datasets
- A list of figures that have associated raw data
- A description of any restrictions on data availability

Data for this manuscript are available upon request, without restrictions. There are no large computational datasets or raw sets of data used for analysis.

Field-specific reporting

Please select the best fit for your research. If you are not sure, read the appropriate sections before making your selection.

☒ Life sciences ☐ Behavioural & social sciences ☐ Ecological, evolutionary & environmental sciences

For a reference copy of the document with all sections, see nature.com/authors/policies/ReportingSummary-flat.pdf

Life sciences study design

All studies must disclose on these points even when the disclosure is negative.

Sample size	No sample sizes were assessed for experimental goals (e.g., no animals/humans).
Data exclusions	No data were excluded from analyses.
Replication	All experiments were conducted 2 or 3 times (replicates) with similar or nearly identical findings. In most cases, two different lab members (co-first authors) conducted the same or similar experiments to verify findings.
Randomization	The cell culture design of all experiments in this report did not necessitate randomization.
Blinding	The cell culture design of all experiments in this report did not necessitate blinding of investigators.

Reporting for specific materials, systems and methods

Materials & experimental systems

n/a	Involved in the study	
☒	☐	Unique biological materials
☐	☒	Antibodies
☐	☒	Eukaryotic cell lines
☒	☐	Palaeontology
☒	☐	Animals and other organisms
☒	☐	Human research participants

Methods

n/a	Involved in the study	
☒	☐	ChIP-seq
☒	☐	Flow cytometry
☒	☐	MRI-based neuroimaging

Antibodies

Antibodies used	Antibodies are rabbit polyclonal or mouse monoclonal (mAb), unless stated. From Santa Cruz Biotech: anti-Psf2 (sc-98556, lotB0409), mAb anti-Cdc45 (IB after co-IP; sc-55569, cloneG12, lotB0317), anti-HBO1 (sc-25379, lot1813), anti-Tip60 (IF; sc-25378), mAb anti-Tip60 (IB; sc-166323, cloneC7, lotH2316), mAb anti-ER (IF and IB; sc-8002, cloneF10, lotJ0716), mAb anti-Mcm7 (sc-9966, clone141.2, lotI0416), mAb anti-H3AcK9/14 (IB; sc-518011, cloneD4, lotJ3117), mAb anti-MCT1(sc-365501). From Sigma: mAb anti-Actin (A5316, cloneAC74), mAb anti-Flag (F3165, cloneM2). From Cell Signaling: rat monoclonal anti-RPA32 (cat2208, clone4E4, lot2), anti-Myc (IP; cat9402, lot11), rabbit monoclonal anti-ER (IP; cat8644, cloneD8H8, lot4), rabbit monoclonal anti-GCN5 (cat3305, cloneC26A10, lot4), anti-TRRAP (cat3966, lot2), rabbit monoclonal anti-H3AcK9 (cat9649, cloneC5B1, lot11), rabbit monoclonal anti-H4AcK12 (cat13944, cloneD2W60, lot1), mAb anti-H4 (cat2935, cloneL64C1, lot6), rabbit monoclonal anti-pH2AX (cat9718, clone20E3, lot10). From Millipore: anti-H1P (cat06597, lot2892887), mAb anti-LacI (cat05503, clone9A5). From Stratagene: rabbit polyclonal anti-LacI (discontinued, but sample available upon request). From Covance: mAb anti-HA (MMS-101R, clone16B12, lot14831802). From Life Technologies: mAb anti-BrdU (B35132, cloneM0BU1, lot1626608). Anti-Myc (IB) was provided by Dr. Steve Hann [Vanderbilt University]; chicken polyclonal anti-Cdc45, anti-Mcm2, and anti-Mcm4 were generated by our group and validated as described (see ref in next section).
Validation	All antibodies, regardless of whether they are commercially derived, are tested in validation experiments against human, mouse, and/or CHO (Chinese hamster ovary) total cell lysates. Results are compared to commercial specification photographs or references given by the manufacturer. In many cases, antibodies are tested against a lysate containing an ectopically-expressed identical version of the antigen that is fused to a larger tag (eg, LacI, ER, or GST tag) that shifts the ectopic protein and allows conclusive evidence to be obtained that the antibody does react with the expected antigen in total lysates. Our methods have been published demonstrating this thorough approach to antibody validation prior to further experimentation by our group (Wong, et al., PlosONE, 2011; 6:1-16; and Supplementary Figures). In other cases, antibodies are compared to lysates that have been treated with siRNA against the target antigen to visualize loss of target bands in experiments. Finally, many antibodies are further validated in this report based on whether they display the expected biological patterns (as compared to other reports, commercial photographs, or biological features of antigen) in immunofluorescence assays shown in the paper.

Eukaryotic cell lines

Policy information about cell lines

Cell line source(s)	CHO (A03_1) cells were obtained from Andrew Belmont (University of Illinois, Champaign). MK cells were obtained from Harold Moses (Vanderbilt University). HaCaT cells were obtained from Petra Boukamp (University of Heidelberg, Germany).
Authentication	None of the cell lines were formally authenticated using genomic approaches. However, all cells are cultured nolonger than 6 weeks from thawed samples, and MK and HaCaT lines are routinely tested for effective synchronization and TGF-beta sensitivity. Morphology is monitored regularly, and MK are cultured in low calcium MEM to prevent terminal differentiation. CHO cells are used between passages 12 and 24 only, to prevent loss of HSR from chromosomes, or other unwanted HSR rearrangements. Visual appearance of the HSR helps to confirm the quality of CHO cells in this report.
Mycoplasma contamination	MK and HaCaT were tested for mycoplasma within a few years of this report, but not routinely. CHO were not tested for mycoplasma, but are continually cultured in gentamicin-containing MEM to suppress such bacterial growth. HaCaT are also cultured in gentamicin. MK are sensitive to antibiotics in the medium and are not cultured in MEM with antibiotics.
Commonly misidentified lines (See ICLAC register)	No commonly misidentified cell lines were used in this report.
