# Supplementary material for: Myc-driven chromatin accessibility regulates Cdc45 assembly into CMG helicases
Source: Commun Biol. 2019 Mar 22;2:110. doi: 10.1038/s42003-019-0353-2 (PMC6430796; doi:10.1038/s42003-019-0353-2)
Supplement: Supplementary file 2 — Supplementary Information [file 42003_2019_353_MOESM2_ESM.pdf]

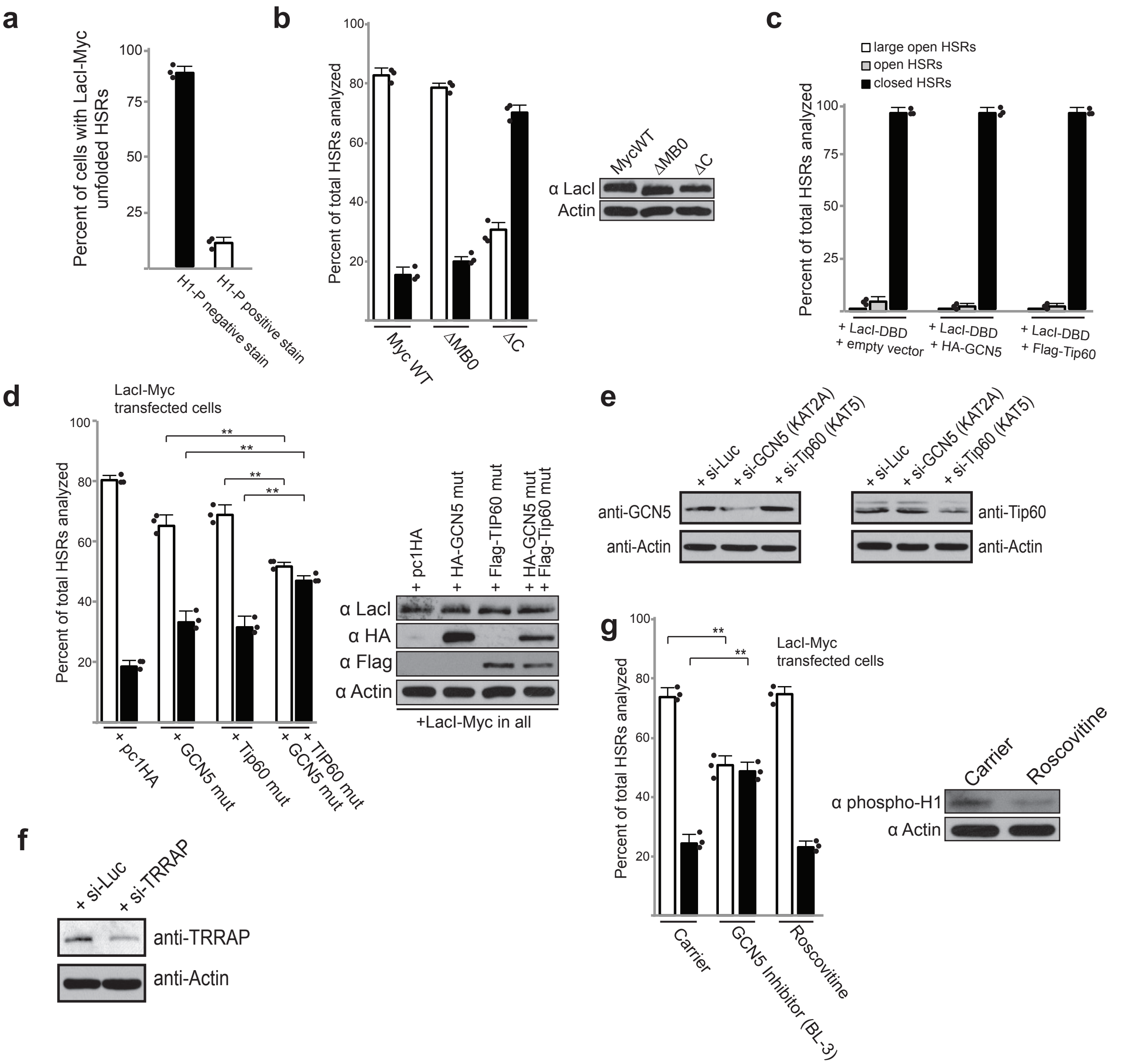

Supplementary Figure 1: Myc-induced unfolding occurs in G1 or early-S, is not dependent on MB0, HAT expression effects on chromatin unfolding at HSRs by LacI-DBD and LacI-Myc, and effects of GCN5 or Cdk2 inhibition on chromatin unfolding.

(A) Chromatin unfolding assays were performed with LacI-Myc, and cells were co-stained for phosphorylated Histone H1. Cells with unfolded HSRs were assessed for high H1-P signal (late-S or G2/M cells) or low/no H1-P signal (G1 or early-S cells). Results from triplicate counts of ~50 transfected cells in each scored field are shown, +/- 1s.d. (B) Chromatin unfolding assays were performed using LacI-wtMyc, LacI-mutMyc (missing MB0), or LacI-mutMyc (ΔC). Additional plasmid was transfected for LacI-mutMyc (ΔC) to create parity in expression with wtMyc. Protein expression is verified in the immunoblot for all LacI derivatives. (C) Chromatin unfolding assays were performed using LacI-DBD transfections (DNA binding domain only of LacI) plus co-expressed GCN5 or Tip60 (or empty vector). Neither enzyme elicits decondensation of the HSRs targeted by LacI-DBD. (D) Chromatin unfolding assays were performed in which LacI-Myc was co-expressed with mutant HAT proteins, individually or together, as indicated. Immunoblots verify that each protein was similarly expressed during the 24 hr transfection prior to HSR analyses. (E) siRNA exposure (using 50-100 nM Smartpools from Dharmacon) of CHO (A03_1) cells partially suppresses protein expression in a specific manner for GCN5 (left) or Tip60 (right). 24 hr transfections were performed and total protein lysates analyzed by immunoblotting. (F) Same experiment as in panel B, but analyzing TRRAP for successful partial knockdown. (G) Chromatin unfolding assays were performed in which LacI-Myc was expressed for 24 hrs, and drugs were added during the last 22 hrs, prior to HSR unfolding analyses. Immunoblot verifies that roscovitine treatment of A03_1 cells could produce partial loss of H1 phosphorylation, as a control for drug exposure. Note in comparison that BL-3 inhibits unfolding, but roscovitine does not.

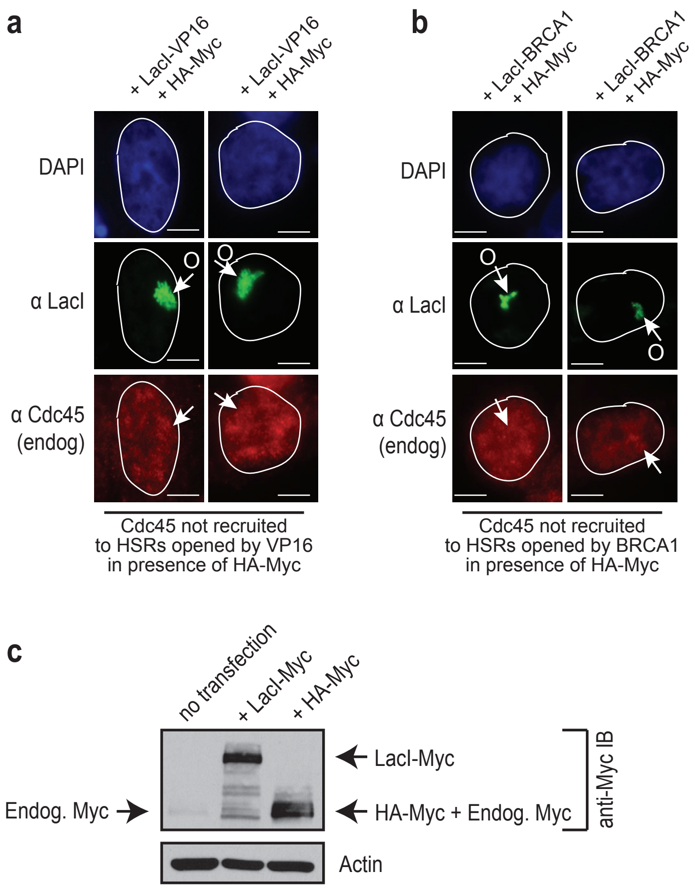

Supplementary Figure 2: Cdc45 is not enriched at HSRs unfolded by LacI-VP16 or LacI-BRCA1 (6c-w) when HA-Myc is co-expressed

(A&B) Chromatin unfolding assays were performed in which HA-Myc was co-expressed alongside LacI-VP16 (A) or LacI-BRCA1(6c-w) (B). Unfolded HSRs were co-stained for LacI and Cdc45. (C) Immunoblot verifying that HA-Myc is expressed at similar levels to LacI-Myc during the transfections. Total lysates were used from similar numbers of plated and transfected cells.

Figure 1a original immunoblots

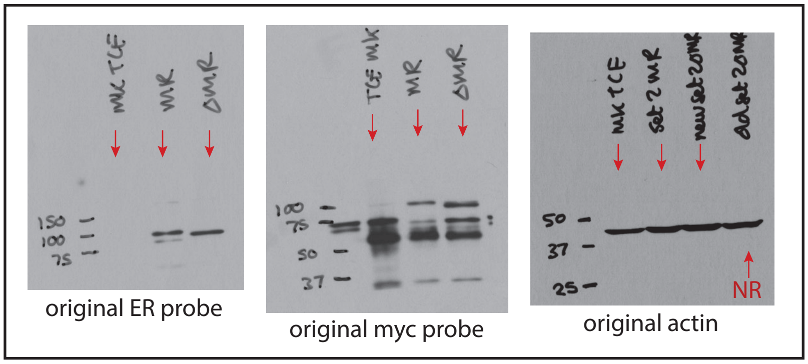

Figure 1g original immunoblots

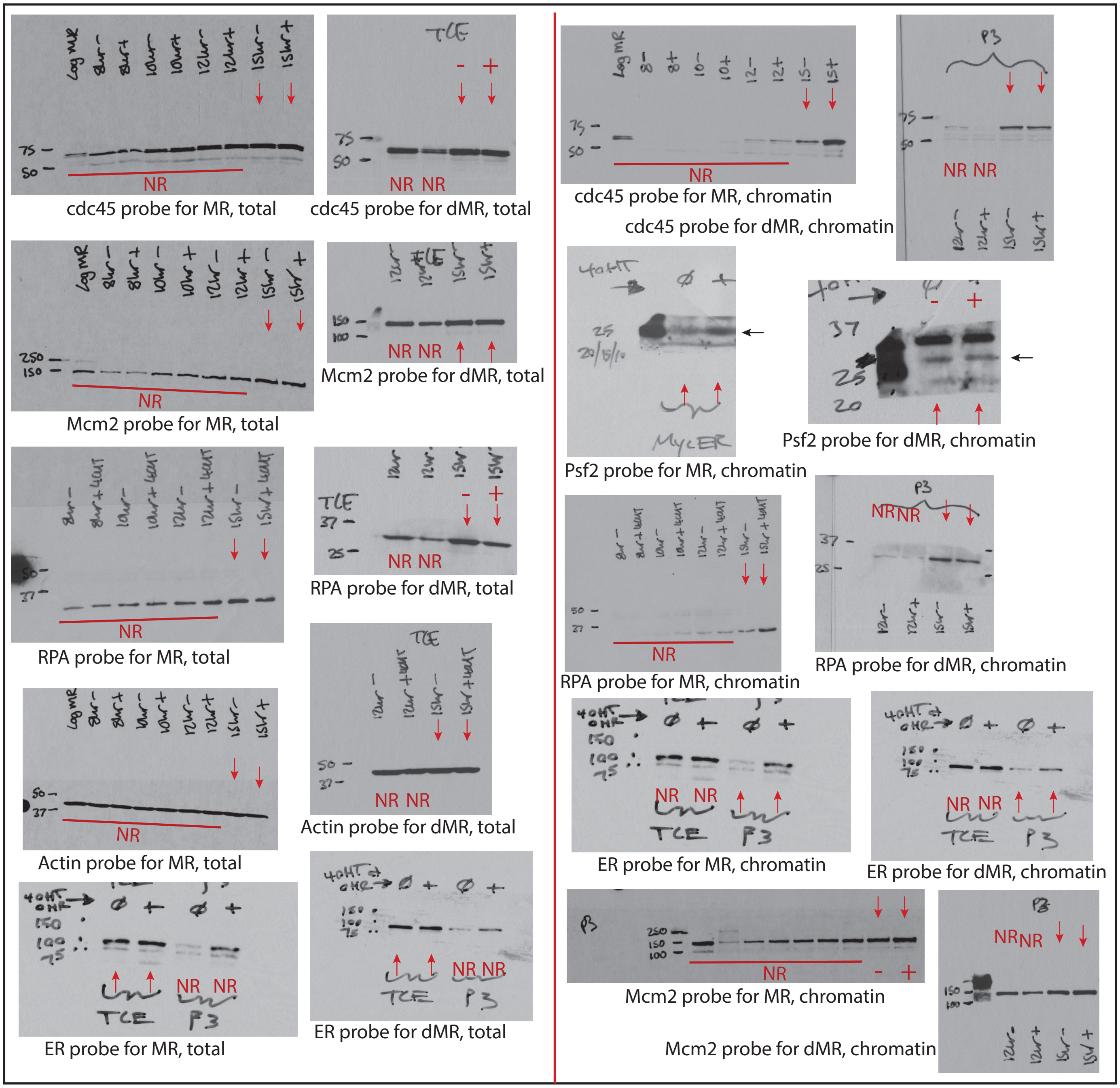

Supplementary Figure 3: Original immunoblots for panels shown in Figure 1A and Figure 1G. Arrows in red indicate lanes used to produce cropped panels in the figures. ‘NR’ indicates lanes not used for the data in the figures, derived from diffferent non-relevant experimental samples. ‘MR’ are samples from MK-MycER cells, ‘dMR’ are samples from MK-deltaMycER cells, ‘TCE’ are samples from total cell extracts, ‘P3’ are samples from chromatin samples. No image alterations were performed on original immunoblots, other than cropping to create the figures in the manuscript.

Figure 1h original immunoblot

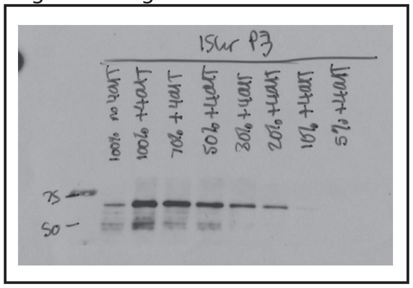

Figure 1i original immunoblots

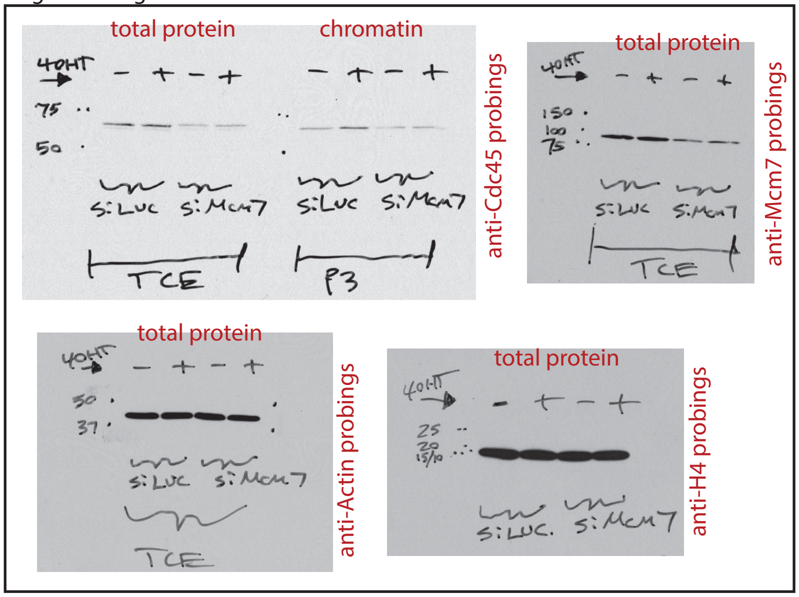

Figure 2g original immunoblots

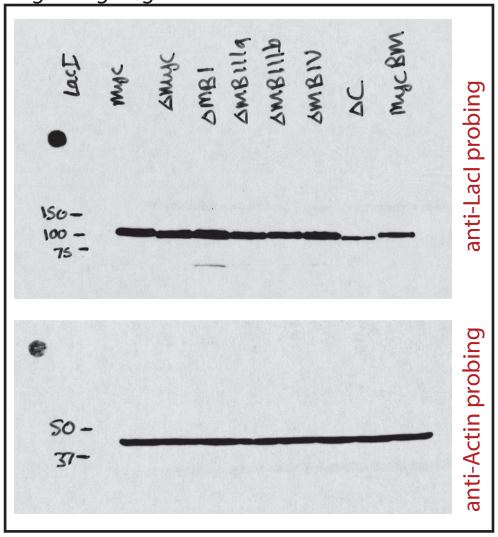

Figure 2i original immunoblots

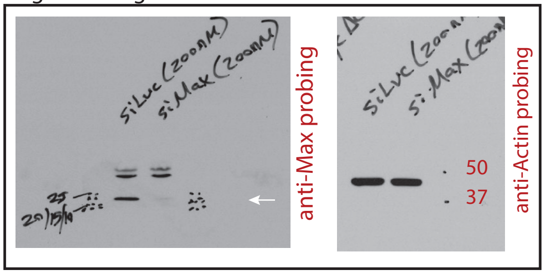

Figure 3b original immunoblots (top half of Panel)

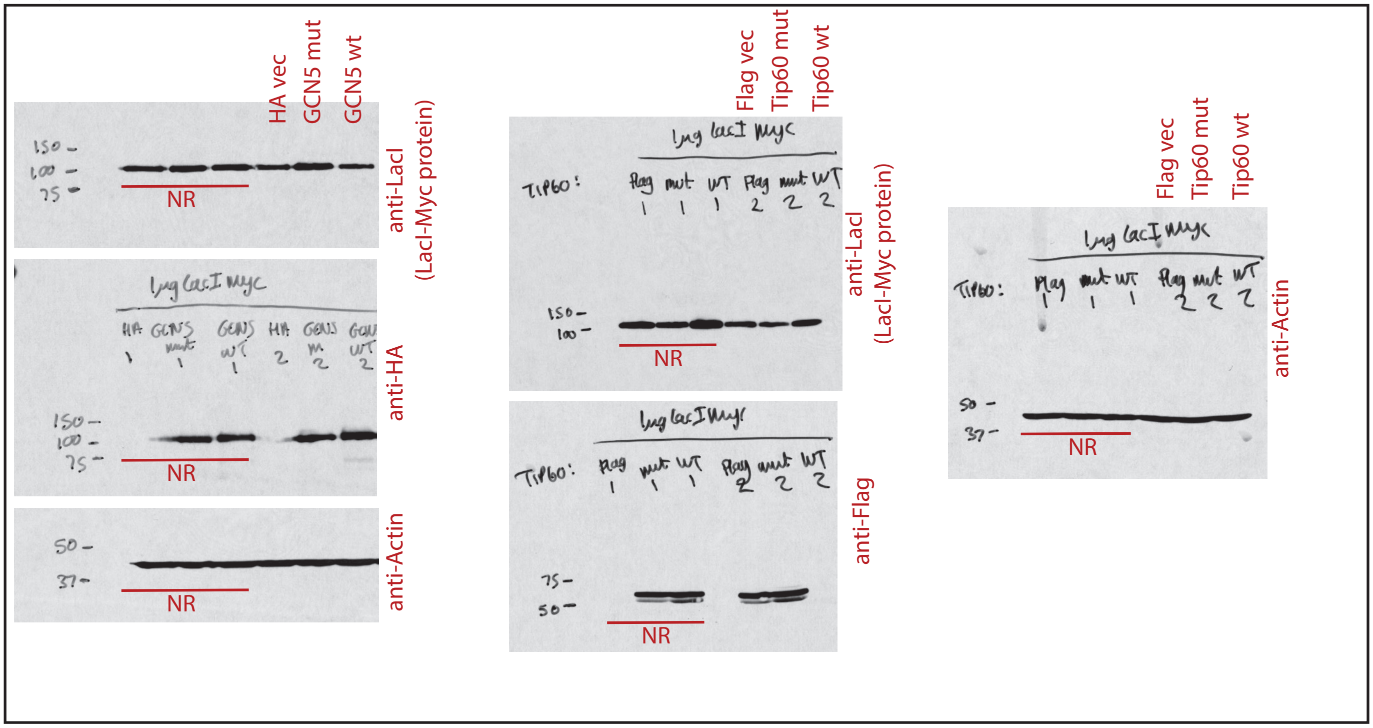

Supplementary Figure 4: Original immunoblots for panels shown in Figures 1H, 1I, 2G, 2I, and 3B. 'NR' indicates lanes not used for the data in the figures, derived from diffferent non-relevant experimental samples. 'TCE' are samples from total cell extracts, 'P3' are samples from chromatin-enriched lysates. No image alterations were performed on original immunoblots, other than cropping to create the figures in the manuscript. 'LacI' refers to LacI-tagged proteins that were expressed, or to anti-LacI immunoblotting.

Figure 3b original immunoblots (bottom half of Panel)

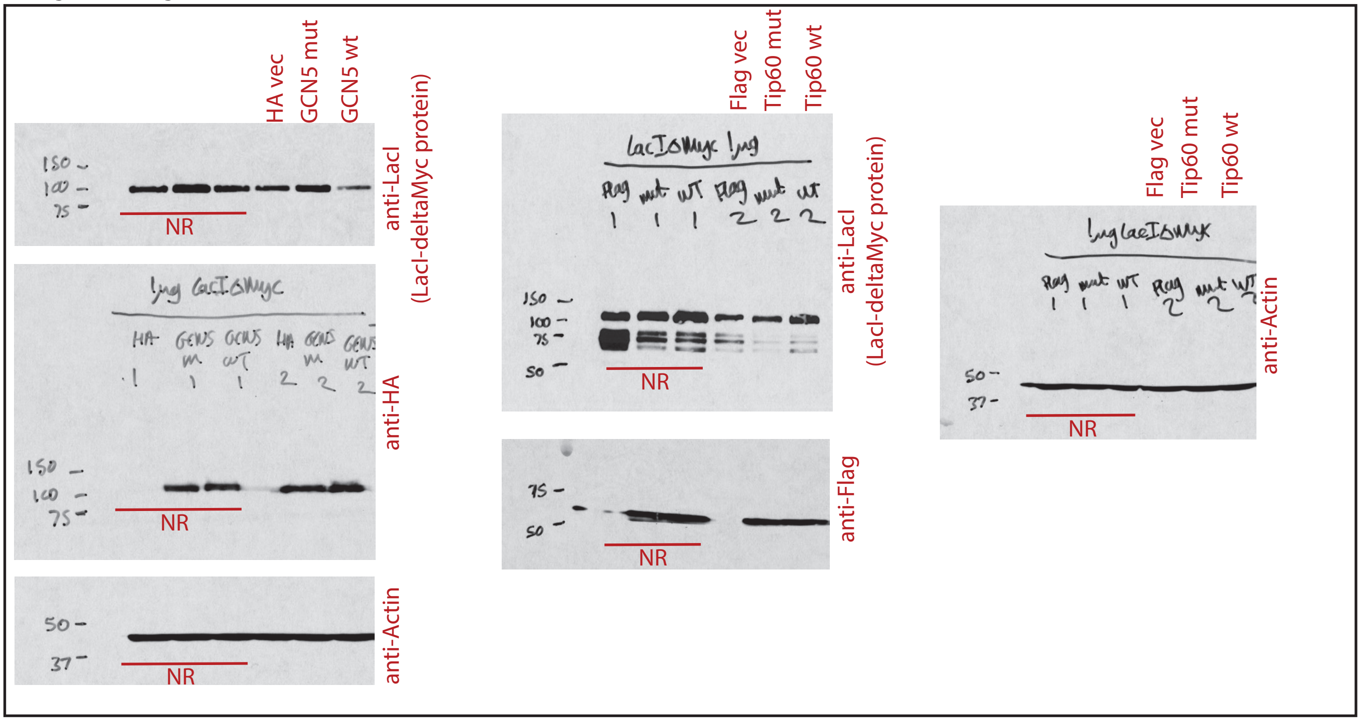

Figure 7a original immunoblot

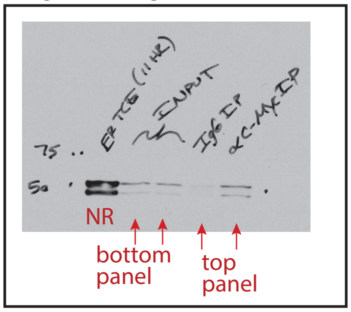

Figure 7b original immunoblots

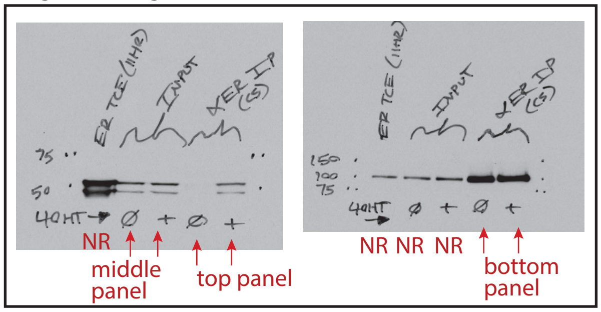

Figure 7d original immunoblots

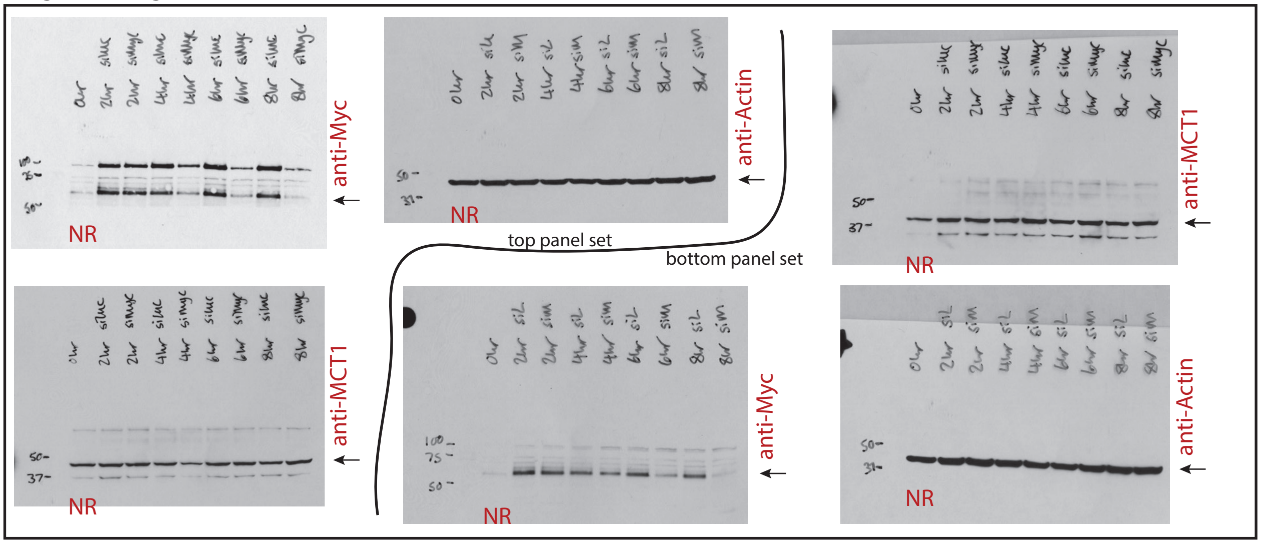

Supplementary Figure 5: Original immunoblots for panels shown in Figures 3B, 7A, 7B, and 7D. Arrows in red indicate lanes used to produce cropped panels in the figures. 'NR' indicates lanes not used for the data in the figures, derived from diffferent non-relevant experimental samples. No image alterations were performed on original immunoblots, other than cropping to create the figures in the manuscript. 'LacI' refers to LacI-tagged proteins that were expressed, or to anti-LacI immunoblotting.

Figure 7e original immunoblots

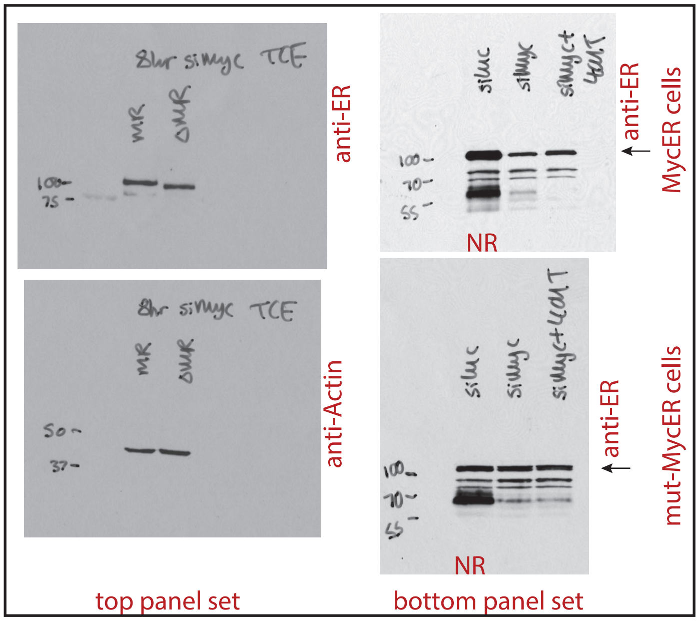

Figure 7g original immunoblots

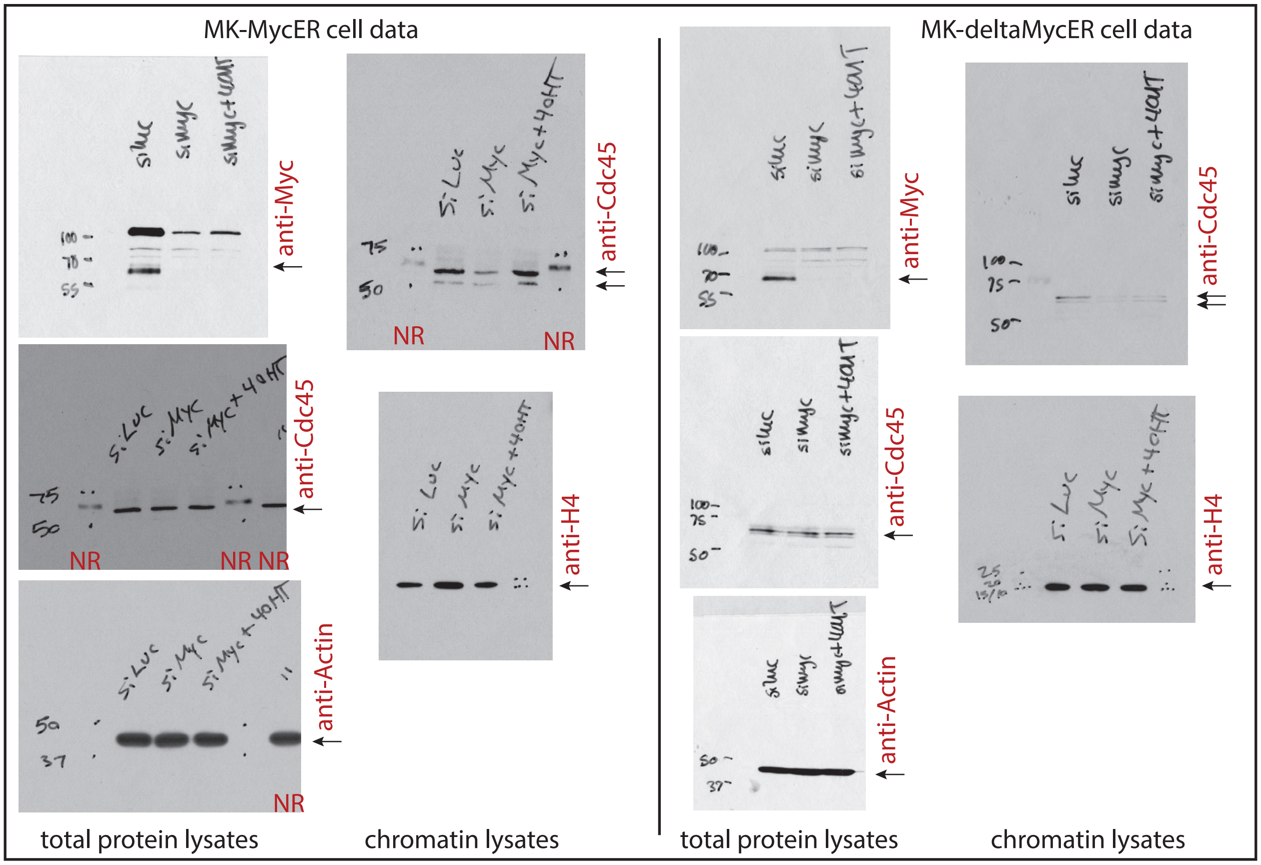

Supplementary Figure 6: Original immunoblots for panels shown in Figure 7E and Figure 7G. 'NR' indicates lanes not used for the data in the figures, derived from diffferent non-relevant experimental samples. No image alterations were performed on original immunoblots, other than cropping to create the figures in the manuscript. 'LacI' refers to LacI-tagged proteins that were expressed, or to anti-LacI immunoblotting.

Figure 8a original immunoblots

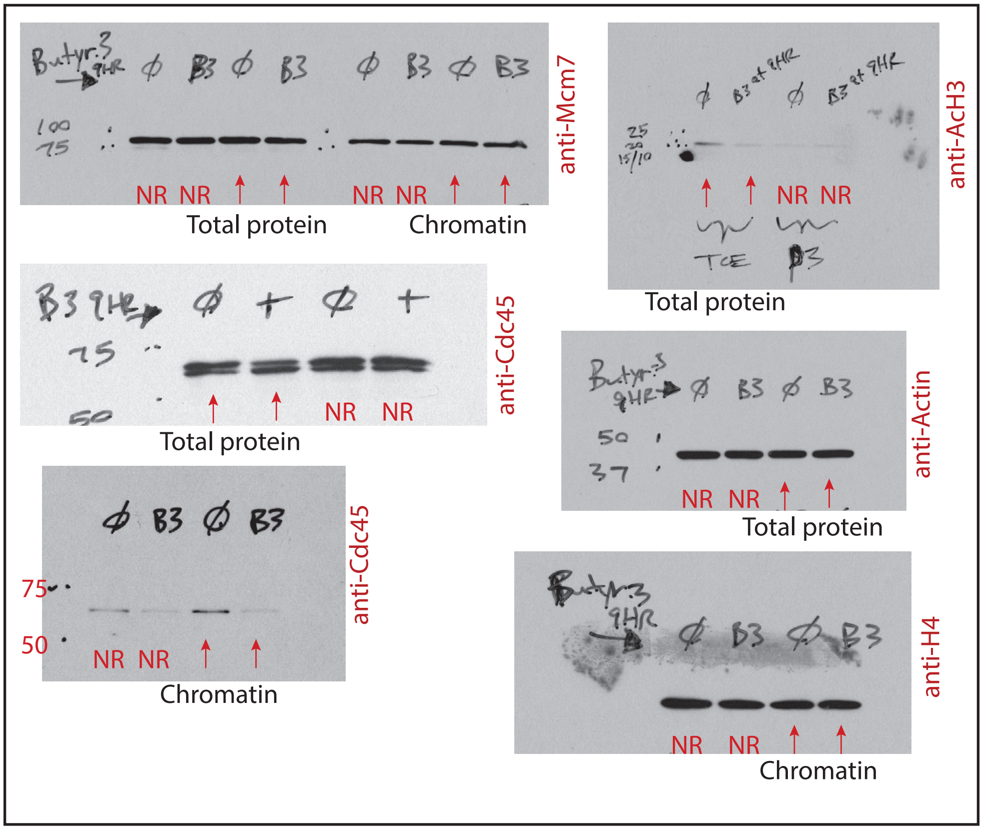

Figure 8b original immunoblots

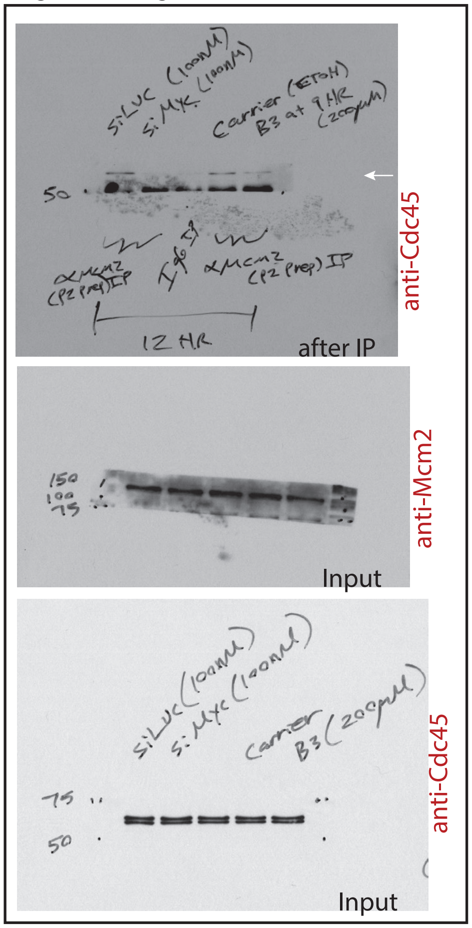

Figure 8c original immunoblots

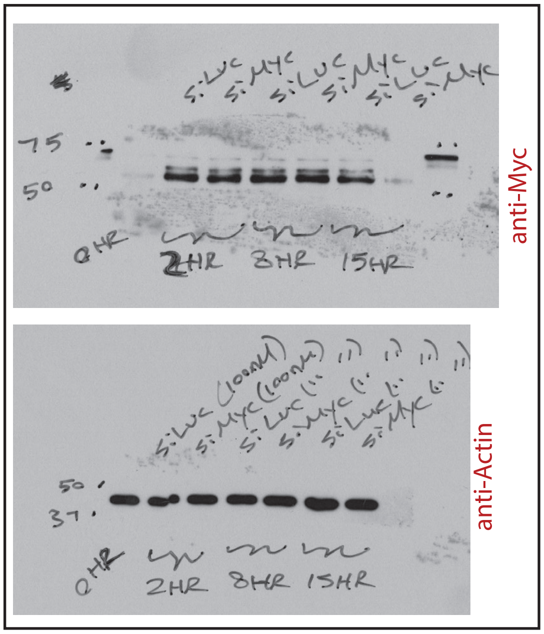

Figure 8d original immunoblots

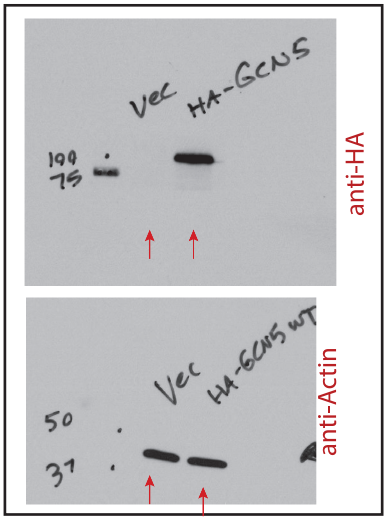

Supplementary Figure 7: Original immunoblots for panels shown in Figures 8A, 8B, 8C, and 8D. 'NR' indicates lanes not used for the data in the figures, derived from diffferent non-relevant experimental samples. Arrows in red indicate lanes used to produce cropped panels in the figures. No image alterations were performed on original immunoblots, other than cropping to create the figures in the manuscript. 'B3' refers to butyrolactone-3 (BL3) GCN5 inhibitor.

Supplementary Figure 1b
original immunoblots

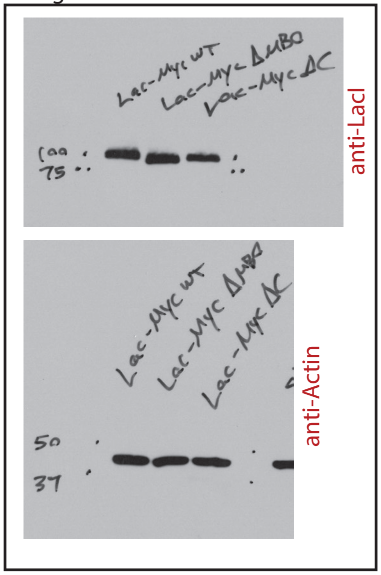

Supplementary Figure 1d
original immunoblots

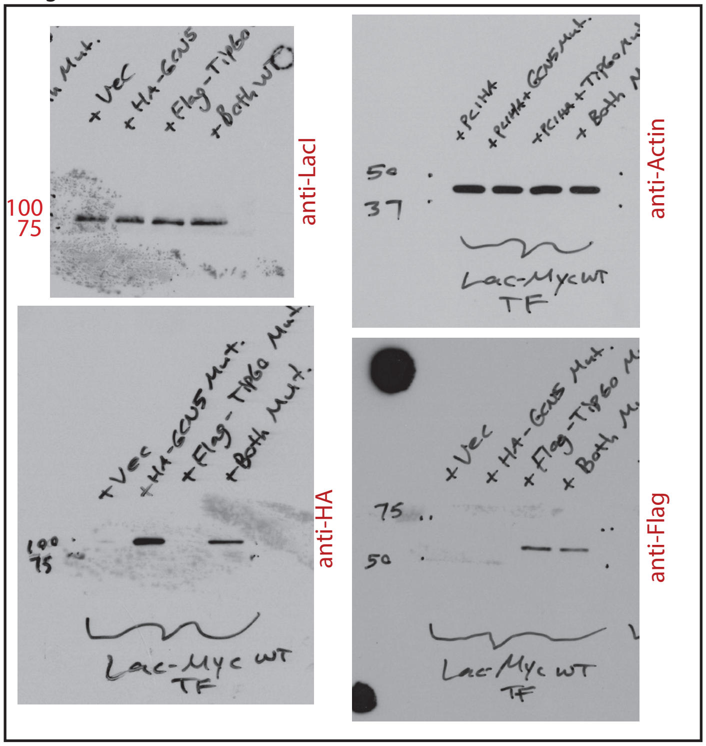

Supplementary Figure 1g
original immunoblots

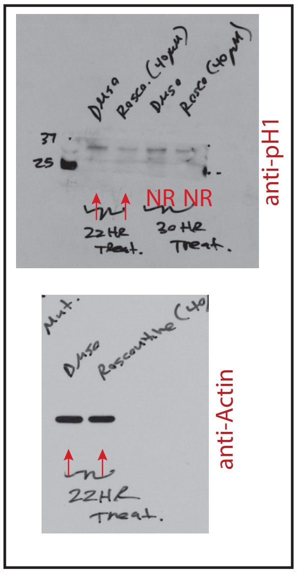

Supplementary Figure 1e &1f
original immunoblots

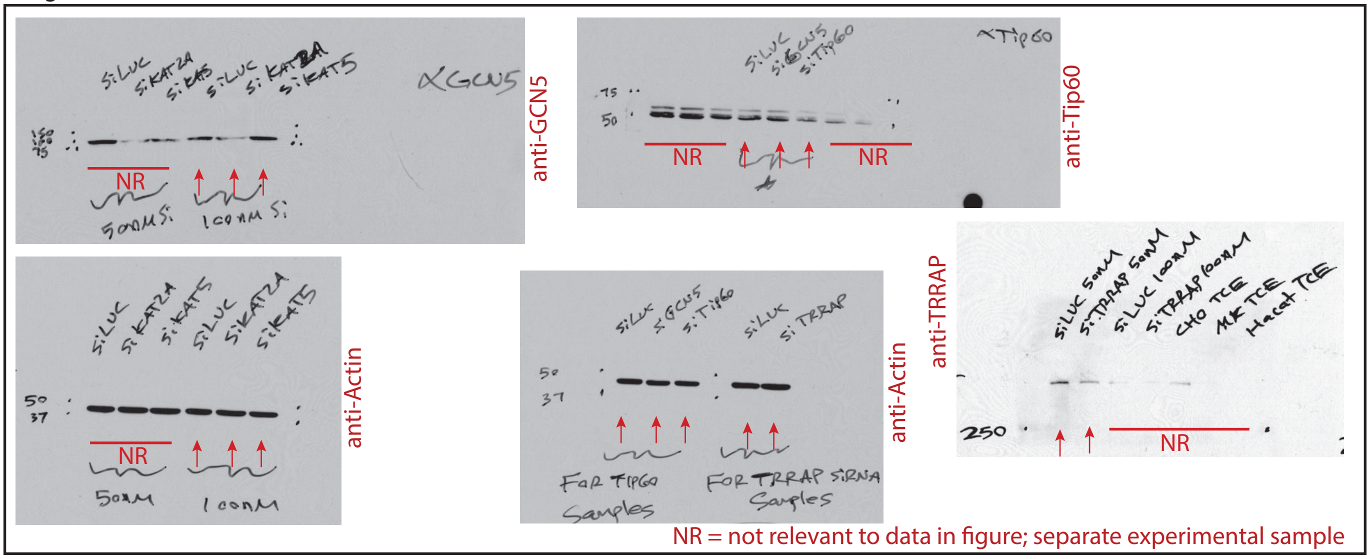

Supplementary Figure 2c
original immunoblots

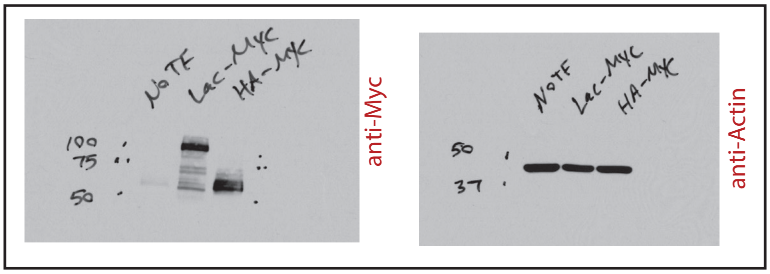

Supplementary Figure 8: Original immunoblots for panels shown in Supplementary Figures 1&2. Arrows in red indicate lanes used to produce cropped panels in the figures. 'NR' indicates lanes not used for the data in the figures, derived from diffferent non-relevant experimental samples. No image alterations were performed on original immunoblots, other than cropping to create the figures in the manuscript. 'LacI' refers to LacI-tagged proteins that were expressed, or to anti-LacI immunoblotting. Kat2A is GCN5, and Kat5 is Tip60. 'Luc' refers to Luciferase.

Protein/BrdU assessed for overlap	with MycER or ∆MycER	Average Co-localization Rate	Average Pearson coefficient
GCN5	MycER	46.6% (± 3.4%)	0.78
TIP60	MycER	43.4% (± 0.5%)	0.72
H4-AcK12	MycER	41.1% (± 0.8%)	0.69
H3-AcK9	MycER	42.4% (± 0.5%)	0.66
HBO1	MycER	14.2% (± 1.2%)	0.38
Mcm2	MycER	26.3% (± 1.0%)	0.52
Cdc45	∆MycER	14.9% (± 1.5%)	0.38
Cdc45	MycER	54.1% (± 1.3%)	0.82
Psf2	MycER	54.8% (± 0.3%)	0.72
RPA (early-S)	MycER	54.4% (± 1.8%)	0.90
RPA (mid-S, later)	MycER	23.2% (± 1.4%)	0.59
BrdU (early-S)	MycER	54.6% (± 1.3%)	0.91
BrdU (mid-S, later)	MycER	22.3% (± 1.4%)	0.60

Supplementary Table 1: Objective quantification of immunofluorescent co-localization rates between MycER and indicated proteins or BrdU

In all experiments, synchronized MK cells were pre-extracted with detergent buffer to assess only those proteins that were tightly-bound to chromatin. MycER or ∆MycER was activated in early-G1 and samples were fixed in early-S (15 hrs). Due to the poisson distribution of the population, some cells were also in mid-S or late-S at time of fixation. Co-localization between MycER (using anti-ER) and specific proteins (using antibodies to indicated protein) was assessed in multiple fields of 80-100 cells using Leica Application Suite X (Leica Microsystems CMS GmbH) software analysis. RPA and BrdU co-localization were assessed by analysis of 10 randomly chosen cells from early-S or mid/late-S (see below). Co-localization Rate is defined as Co-localization Area (red and green pixels overlapping)/Area Foreground. Area Foreground is defined as Area of Image (pixels with signal)/Area of Background (no-signal pixels). Averages +/- 1s.d. are shown in the Table for each category, along with average Pearson correlation coefficients for each. Note that in the cases of RPA and BrdU, focal patterns for each of the prior were also used to determine the relative stage of nuclei within S-phase. For example, early-S nuclei display a widely-distributed focal and punctate pattern across the nuclear interior, while mid-S tend to display a nuclear peripheral staining pattern. Such criteria assigned the majority of co-localization between MycER and RPA or BrdU to early-S stage cells.
